# Scintillating and Photoluminescent Ratiometric and Visual Luminescence Thermometry Based on the Ce^3+^-Doped Eutectic Structures

**DOI:** 10.1021/acsami.5c16426

**Published:** 2025-10-15

**Authors:** Karol Bartosiewicz, Maja Szymczak, Masao Yoshino, Takahiko Horiai, Robert Tomala, Justyna Zeler, Aleksandra Owczarek, Damian Szymanski, Marcin E. Witkowski, Vítězslav Jarý, Winicjusz Drozdowski, Eugeniusz Zych, Akira Yoshikawa, Łukasz Marciniak

**Affiliations:** † 86889 Institute of Physics, Czech Academy of Sciences, Na Slovance 1999/2, Praha 18200, Czechia; ‡ Institute of Low Temperature and Structure Research, 215275 Polish Academy of Sciences, Okólna 2, Wrocław 50422, Poland; § New Industry Creation Hatchery Center, 426647 Tohoku University, 2-1-1 Katahira Aoba-ku, Sendai, Miyagi 980-8577, Japan; ∥ 73459 National Institute of Advanced Industrial Science and Technology (AIST), Core Electronics Technology Research Institute, AIST Tsukuba Central 5, 1-1-1 Higashi, Tsukuba, Ibaraki 305-8565, Japan; ⊥ Faculty of Chemistry and Geosciences, Vilnius University, Naugarduko g. 24, Vilnius 03225, Lithuania; # 49572 University of Wrocław, Faculty of Chemistry, 14 F. Joliot-Curie Street, Wrocław 50383, Poland; ∇ Institute of Physics, Faculty of Physics, Astronomy and Informatics, 49577 Nicolaus Copernicus University in Toruń, Grudziądzka 5, Toruń 87100, Poland; ○ Institute for Materials Research, 84063 Tohoku University, 2-1-1 Katahira Aoba-ku, Sendai, Miyagi 980-8577, Japan

**Keywords:** luminescence thermometry, scintillation thermometry, eutectic, crystal, garnet, perovskite, high-power white light phosphor, thermoluminescence

## Abstract

A next-generation class of dual-phase, multifunctional photoconversion and thermal sensing materials has been developed using Ce^3+^-doped YAG-YAP eutectic crystals, synthesized via directional solidification at variable rates (0.1–0.9 mm/min) to precisely tailor phase morphology and dopant distribution. Structural and compositional analyses revealed a lamellar microstructure comprising alternating garnet (Y_3_Al_5_O_12_, YAG) and perovskite (YAlO_3_, YAP) domains, with Ce^3+^ ions preferentially partitioned into the garnet phase at elevated solidification rates. Systematic control of domain sizes was achieved by modulating the growth rate. Slower growth resulted in larger domains that enabled near-complete transmission of blue light through YAP, whereas faster growth produced finer structures that led to increased scattering and absorption of blue light. This morphology-driven optical tunability enabled dynamic control over the correlated color temperature (CCT), ranging from cool to warm white emissions. Beyond structural engineering, the eutectics demonstrated dual-mode thermal sensing via ratiometric luminescence thermometry under both photoluminescence (PL) and X-ray-induced scintillation excitation. Excitation modality significantly affected thermal sensitivity due to distinct charge transport and energy transfer dynamics. Under PL, the relative sensitivity reached 0.47% K^–1^, while scintillation-based excitation achieved an enhanced sensitivity up to 1.1% K^–1^. Crucially, the scintillation mode permits passive, remote temperature monitoring without external optical excitation, activated solely by ambient ionizing radiation. These capabilities position Ce^3+^-doped YAG-YAP eutectics as promising candidates for advanced thermal sensing in extreme environments, including nuclear reactors, aerospace systems, and high-energy particle detectors.

## Graphical abstract

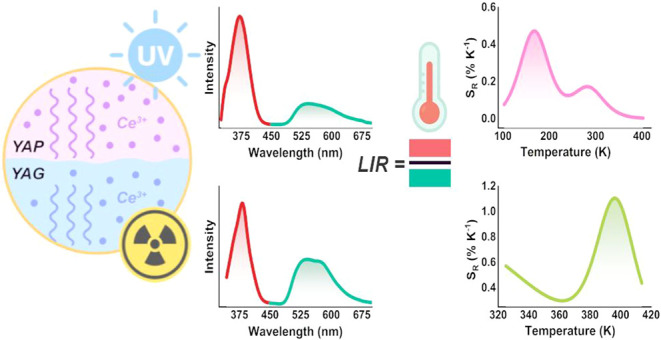

## 1 Introduction

Composite materials are widely studied for their ability to integrate distinct physical and chemical characteristics of individual components into a single material system, resulting in enhanced mechanical and thermal performance as well as tunable functional characteristics such as electrical, optical, or catalytic behavior.^1–8^ Within the broad category of composites, eutectic materials constitute a unique and important class. They are characterized as intimate, single-phase mixtures of two or more constituents whose combined melting point is lower than that of any individual component.^1–4^ The eutectic composition solidifies at a single, distinct temperature called the eutectic temperature. The formation of eutectic materials occurs through a process of cooperative solidification, where the constituent phases grow together from the liquid melt as a diffusion couple. At the eutectic point, a unique invariant reaction takes place, where the liquid transforms directly into two or more solid phases simultaneously. This simultaneous solidification is a key characteristic distinguishing eutectic systems from other alloy systems, in which individual components typically solidify over a range of temperatures rather than at a single point.^9^ Such behavior enables eutectic systems to exhibit highly uniform and controllable microstructures, making them valuable for applications requiring precise thermal, mechanical, or optical performance. Eutectic microstructures form in diverse morphologies, governed by solidification conditions and interfacial energy dynamics between the constituent phases. The geometrical configurations include regular lamellar and rod-like structures and more complex morphologies, such as irregular, broken-lamellar, spiral, quasi-regular, and globular structures. These diverse morphologies significantly influence the resulting material properties, including mechanical strength, thermal conductivity, and optical properties.^10^ The binary Al_2_O_3_–Y_2_O_3_ oxide system can form up to four distinct eutectic composite crystals, depending on the specific molar ratio between Al_2_O_3_ and Y_2_O_3_. Among these, the Y_3_Al_5_O_12_–Al_2_O_3_ (YAG–Al_2_O_3_) eutectic system has been the most extensively investigated. In particular, Ce^3+^-doped YAG-Al_2_O_3_ eutectics have attracted significant interest as efficient phosphor materials for high-power white LEDs.^6–8^ These eutectics offer enhanced luminous efficacy, thermal stability, and mechanical strength compared to their single-phase Ce^3+^-doped YAG.^11^ However, due to the fact that Ce^3+^ cannot be introduced into the Al_2_O_3_ structure, the presence of this phase in Ce^3+^-doped YAG-Al_2_O_3_ eutectics enables only improvement in the mechanical properties of the eutectics.

This study provides the detailed characterization of the second eutectic composite phase identified in the binary Al_2_O_3_–Y_2_O_3_ system, focusing on the Y_3_Al_5_O_12_–YAlO_3_ (YAG-YAP) eutectic structure, which advances the understanding of phase equilibria and microstructural evolution in high-temperature oxide systems. This composite was grown from the melt via unidirectional solidification, resulting in a lamellar structure with two distinct garnet and perovskite phases. Owing to the different crystallographic environments and local crystal fields within YAG and YAP phases, the Ce^3+^ ions incorporated into each phase exhibit distinct photoluminescence characteristics. Specifically, Ce^3+^ emission band is observed at around 370 nm in the YAP phase,^12^ while in the YAG phase, it shifts to approximately 520 nm.^12^ Notably, the excitation bands of Ce^3+^ in both phases overlap, allowing simultaneous excitation of Ce^3+^ ions across both lattices.^13,14^ This spectral behavior not only enables a broadening of the spectral range in which Ce^3+^ luminescence, but more importantly, it exploits the distinct thermal behavior of Ce^3+^ emission in each of the constituent phases to facilitate the construction of ratiometric luminescent temperature sensors. Given that both emission bands fall within the UV–visible region of the spectrum, temperature readout using Ce^3+^-doped Y_3_Al_5_O_12_–YAlO_3_ eutectic structure can be achieved not only through analysis of the luminescence intensity ratio but also via colorimetric evaluation of the emitted light.^12,15^ Furthermore, both Ce^3+^-doped YAG and YAP are well-established scintillators operating in the ns time range with high density and subnanosecond scintillation response times.^15,16^ The integration of both phases within a single eutectic structure paves the way for its application in scintillation thermometry under radiation-intensive conditions, requiring remote temperature monitoring. An additional benefit of the YAG-YAP eutectic is its very good thermal conductivity,^17^ which promotes uniform heat distribution across the material, thereby enhancing measurement precision and thermal management under operational conditions. Furthermore, the characteristic mechanism of eutectic solidification facilitates the formation of a homogeneously distributed microstructure, in which the two immiscible constituent phases codevelop into an interpenetrating framework, ensuring uniform phase integration throughout the entire crystalline matrix.^1,8^

In this study, Ce^3+^-doped YAG-YAP eutectic crystals are investigated as a model system for investigating fundamentally distinct excitation mechanisms of Ce^3+^ ions and their influence on the temperature sensitivity in luminescence thermometry. The first excitation mechanism involves direct optical excitation into the 4f → 5d absorption bands of Ce^3+^ ions present in both the garnet and the perovskite phases of the eutectic composite. In this case, incident photons are absorbed directly by the Ce^3+^ centers, promoting electrons from the ground 4f state to the excited 5d state. In this excitation mechanism, the temperature effects are largely governed by intraionic processes such as thermal population of higher excited states, nonradiative relaxation, and phonon-assisted transitions. The second mechanism operates through a scintillation process, wherein excitation occurs via the host crystal’s electronic band structure. High-energy photons or particles generate electron–hole pairs in the valence and conduction bands of the host material. These charge carriers subsequently migrate through the lattice and are eventually captured by Ce^3+^ ions, which serve as luminescence centers. This indirect mechanism encompasses a more complex chain of events, including carrier transport, energy transfer, and interactions with structural defects. A key factor influencing the scintillation excitation pathway is the presence of trapping centers within the host lattice. These defect states can transiently capture migrating charge carriers, delaying or inhibiting their recombination at the Ce^3+^ sites. The thermal activation and release of trapped carriers introduce additional temperature dependencies in the luminescence behavior, affecting both the intensity and the kinetics of the emitted light. These findings contribute to the fundamental understanding of excitation-dependent thermal sensitivity in rare-earth-activated phosphors while advancing the development of next-generation luminescence thermometry phosphors with enhanced sensitivity and operational versatility. Further in this study, a comprehensive thermometric characterization of Ce^3+^ doped YAG-YAP eutectics synthesized at various crystal growth rates was performed in order to enhance their applicability in industrial environments. The results demonstrated that the crystallization process can be accelerated by a factor of 9 without compromising the material’s thermometric performance. Remarkably, faster growth preserved the luminescent sensing capabilities, both as a scintillating and photoluminescent thermometer and also led to improved structural homogeneity, further supporting the material’s suitability for scalable production and real-world thermal monitoring applications.

## 2 Methodology

### 2.1 Composite Eutectic Crystal Growth

The Ce^3+^-doped YAG-YAP eutectic composite crystals, along with Ce^3+^-doped YAG and YAP single crystals, were grown from the melt using the micropulling-down (μ-PD) technique.^18,19^ High-purity oxide powders of Y_2_O_3_ (99.99%), Al_2_O_3_ (99.99%), and CeO_2_ (99.99%) (Iwatani Corporation) were used as starting materials. The oxides were accurately weighed based on the Y_2_O_3_–Al_2_O_3_ binary phase diagram, targeting a eutectic molar composition of 45.5 mol % Y_2_O_3_ and 54.5 mol % Al_2_O_3_. To introduce Ce^3+^ ions, CeO_2_ was added in an amount corresponding to 0.5 mol % relative to the total molar quantity of Y_2_O_3_. The unmixed oxides were loaded into an iridium crucible. Crystal growth was conducted by pulling the melt through a capillary channel located at the bottom of the crucible, which featured a 3 mm outer diameter and a 0.4 mm nozzle. A <111>-oriented YAG single crystal was used as the seed. The eutectic crystals were grown at pulling rates of 0.1, 0.6, and 0.9 mm/min to investigate the influence of growth rate on microstructural development. The Ce^3+^-doped YAG and YAP single crystals were grown at a pulling rate of 0.1 mm/min. The growth was performed under a N_2_ protective atmosphere. The flowing N_2_ gas with a pressure of 1.04 atom was used during the solidification process.

### 2.2 PXRD, SEM–EDS, and EPMA Analysis

Fragments of the grown crystals were mechanically pulverized into a fine powder using an agate mortar. Phase identification and crystallographic characterization were performed by using powder X-ray diffraction (PXRD) on a BRUKER D8 DISCOVER-HS diffractometer. Data were collected over a 2θ range of 20 to 65° with a step size of ∼0.01°, ensuring high angular resolution for accurate structural analysis. Cu Kα radiation (λ ≈ 1.54 Å, photon energy *E* = 8.05 keV) was used as the X-ray source. Rietveld refinement was performed using Jana2020 software.^20^ The experimentally obtained phase fractions were compared with theoretical values predicted by the lever rule,^21^ according to eq 1:

1 $$f_{YAG}=\frac{C_{YAP}-C_{0}}{C_{YAP}-C_{YAG}},\;f_{YAP}=\frac{C_{o}-C_{YAG}}{C_{YAP}-C_{YAG}}$$

where *C*_0_ denotes the initial composition expressed in terms of mol % Y_2_O_3_, while *C*_YAG_ and *C*_YAP_ correspond to YAG and YAP composition, respectively. Microstructural features and elemental distribution in Ce^3+^-doped YAG-YAP eutectic crystals were evaluated by using a NovaNanoSEM 230 field-emission scanning electron microscope (FE-SEM) integrated with an energy-dispersive X-ray spectroscopy (EDS) detector (Genesis XM4). Prior to SEM imaging, the Ce^3+^-doped YAG-YAP eutectic composite crystals were included in thermosetting Bakelite resin with carbon filter (PolyFast, Struers), carefully polished, and sputter-coated with gold. Secondary electron (SE) signals were acquired at 3.0 kV acceleration voltage for high-resolution topographical imaging, while EDS elemental maps were generated at 30.0 kV to resolve spatial compositional variations. Due to the low concentration of Ce atoms in the examined samples, EDS mapping were not feasible for aforementioned element. Consequently, the EDS map for the Ce element was not included in the subsequent discussion. The quantitative chemical analysis of the eutectics for the Y, Al, Ce, and O contents along the radial direction was performed by the electron probe microanalysis (EPMA, JXA-8530 F, JEOL) equipped with wavelength-dispersive spectrometers (WDS).

### 2.3 Optical, Photoconversion, Luminescence, and Scintillation Characteristics

The absorption spectra were measured with a JASCO V-730 instrument in the 200–800 nm spectral range at 300 K. Photoconversion properties were evaluated by placing the crystalline sample atop a calibrated illumination system incorporating a collimating lens, which adjusted the beam profile to match the geometric dimensions of the specimen. The excitation source consisted of a 450 nm light-emitting diode (LED) operating at a constant optical power of 1.0 W. Emission spectra were recorded by using a Gigahertz BTS-256LED spectrometer integrated with an optical integrating sphere to ensure uniform light collection and accurate spectral analysis. Chromaticity coordinates in the CIE 1931 color space were calculated using the BTS256 Software Suite, version 1.24.6 (Gigahertz-Optik GmbH, Germany), which is specifically designed for spectral data processing and colorimetric evaluation. The luminescence characteristics including emission and excitation spectra, as well as luminescence decay profiles were performed using an FLS1000 Fluorescence Spectrometer (Edinburgh Instruments), equipped with a 450 W xenon lamp. The temperature of the samples during temperature-dependent measurements was controlled by a THMS 600 heating–cooling stage from Linkam with 0.1 °C temperature stability and 0.1 °C set point resolution. Before each measurement, temperature stabilization was carried out for 2 min.

### 2.4 Radioluminescence and Thermoluminescence Measurements

Low-temperature (10–350 K) radioluminescence (RL) and thermoluminescence (TL) measurements were conducted by using a modular cryogenic spectroscopy system. The apparatus comprised an Inel X-ray generator (Cu anode excitation source), an ARC SP-500i high-resolution monochromator, a Hamamatsu R928 photomultiplier tube (PMT), and an APD Cryogenics closed-cycle helium refrigeration unit regulated by a Lake Shore 330 temperature controller. The RL spectra were collected under isochronal cooling from 350 to 10 K to suppress artifacts arising from thermally stimulated charge carrier migration, thereby isolating purely radiation-induced luminescence. The TL glow curves were acquired by irradiating samples for 10 min with X-rays prior to linear heating from 10 to 300 K at a rate of 0.14 K s^–1^. The TL glow curves were modeled using a quasi-continuous trap energy framework. The low-temperature glow curves obtained from thermoluminescence measurements were analyzed by using quasi-continuous distributions of trap levels. In this approach, a single trap does not correspond to a discrete energy value but rather spans a range of energy values, as determined by the number of Gaussian functions used in the fitting process.^22^

High-temperature thermoluminescence and X-ray-excited luminescence measurements, spanning the 300–650 K range, were conducted using a *Lexsyg Research* fully automated TL/OSL reader (Freiberg Instruments GmbH). X-ray excitation was provided by a VF-50J RTG lamp with a tungsten anode, operated under two distinct conditions: 12 kV and 0.1 mA for TL glow curve acquisition, and 45 kV and 0.5 mA for X-ray-excited luminescence measurements. Thermoluminescence glow curves were captured using a 9235QB photomultiplier tube (ET Enterprises) over the temperature range of 303–723 K (30–450 °C), employing a linear heating rate of 5 °C/s. X-ray-excited luminescence spectra were recorded by an Andor DU420A-OE CCD detector, thermoelectrically cooled to 193 K to reduce noise and enhance signal fidelity. Prior to measurement, samples were exposed to X-ray irradiation and subsequently transferred to the reader’s heating stage. No optical filters were applied during spectral collection, and all experimental operations were managed via the *LexStudio 2* software suite.

## 3 Results and Discussion

### 3.1 Crystal Phase and Morphology

The Ce^3+^-doped YAG-YAP eutectic composite crystals were synthesized at three distinct growth rates: 0.1, 0.6, and 0.9 mm/min. Figure 1a presents the corresponding as-grown rods alongside their polished plates, illustrating the high quality and visual uniformity of the samples obtained under varying growth conditions. The rods exhibit a regular cylindrical geometry, indicating successful crystallization of the entire melt. The polished cross sections reveal distinct microstructural features depending on the crystallization rate. At a low growth rate of 0.1 mm/min, a transparent core is observed. In contrast, higher crystallization rates (0.6 and 0.9 mm/min) result in a more homogeneous radial distribution of the Y_3_Al_5_O_12_ garnet (YAG) and YAlO_3_ perovskite (YAP) phases throughout the crystal.

**Figure 1:**
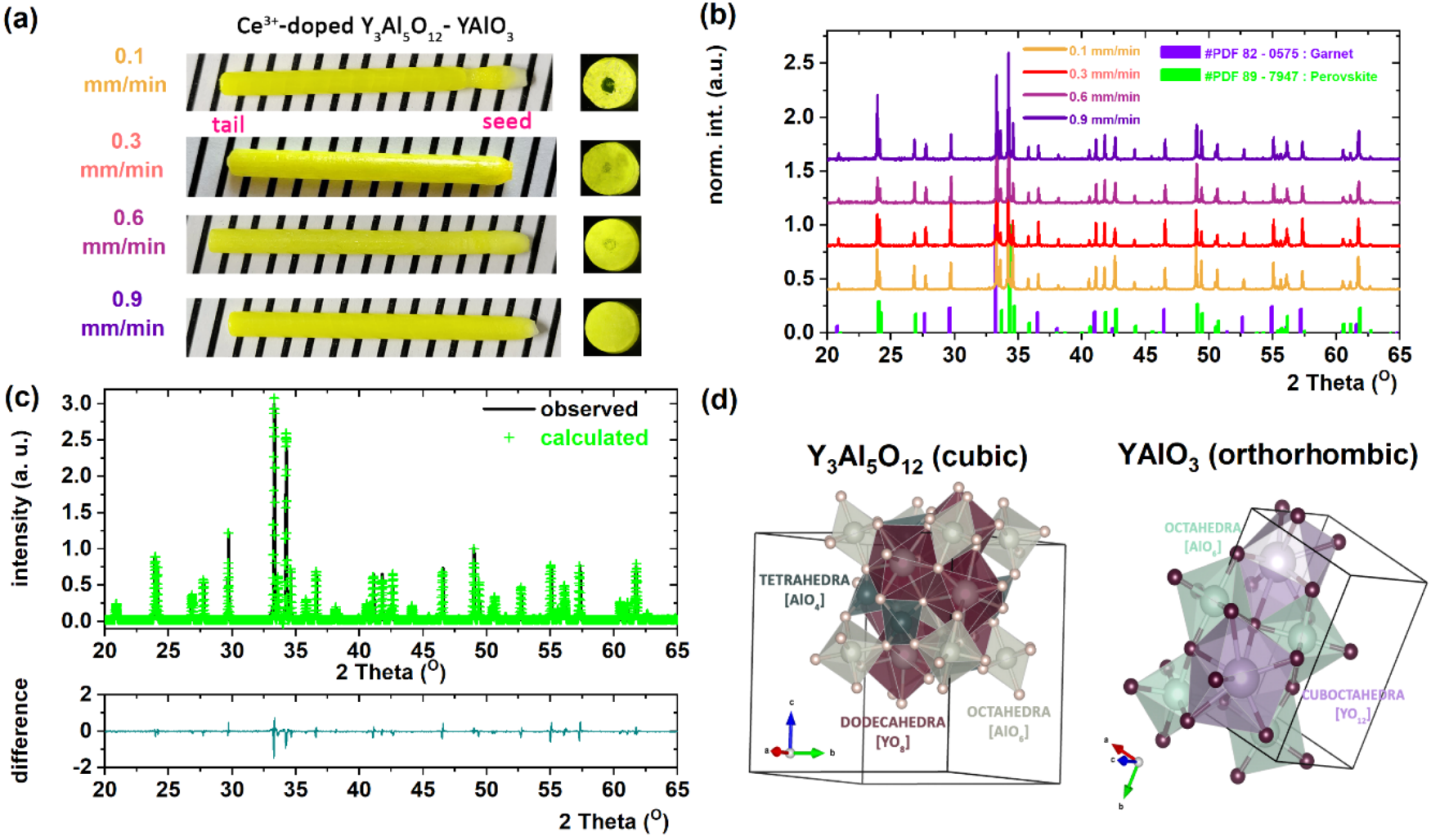
(a) As-grown Ce^3+^-doped YAG-YAP eutectic crystals grown at pulling rates ranging from 0.1 to 0.9 mm/min, alongside radially polished cylindrical samples. (b) Comparison of theoretical and experimentally observed XRD patterns of the YAG-YAP eutectics. (c) Rietveld refinement of the XRD pattern for the Ce^3+^-doped YAG-YAP eutectic crystal grown at a pulling rate of 0.1 mm/min. The curve at the bottom represents the difference between the observed and calculated profiles. (d) Schematic representation of the YAG and YAP unit cells, as obtained from structural Rietveld refinement.

The powder X-ray diffraction patterns of all eutectic crystals confirmed the presence of a eutectic microstructure composed of a mixture of YAG (cubic phase, space group *Ia3d*, No. 230) and YAP (orthorhombic phase, space group *Pnma*, No. 62). To quantify the relative phase fractions within the crystal matrix, Rietveld refinement was carried out for each sample. The results of the refinement, including the garnet and perovskite phase fractions, are presented in Figure 1c and Table 1. The experimentally estimated phase percentage fractions of YAG and YAP are in close agreement with the theoretical values predicted by the lever rule,^21^ which yield approximately 36 ± 4% of YAG and 64 ± 4% of YAP, respectively

**Table 1:** Phase Fractions of YAG and YAP as a Function of the Solidification Rate^a^

	**garnet** (cubic)		**perovskite** (orthorhombic)			
		***a***_***0***_ **(Å)**		***a***_***0***_ **(Å)**		
**rate of solidification** (mm/min)	**phase fraction ± error** (%)	***a* = *b* = *c***	**phase fraction ± error** (%)	***a***	***b***	***c***
0.1	38.6 ± 4	12.072	61.4 ± 4	5.364	7.426	5.275
0.3	37.9 ± 4	12.073	61.7 ± 4	5.365	7.428	5.274
0.6	37.5 ± 4	12.074	62.5 ± 4	5.372	7.414	5.267
0.9	37.1 ± 4	12.081	62.9 ± 4	5.383	7.432	5.290

a The lattice constants *a* = *b* = *c* for the cubic garnet phase and the *a*, *b*, and *c* for the orthorhombic perovskite phase determined by Rietveld refinement.

The radial distribution of YAG and YAP phases as a function of increasing solidification rate is illustrated by the EDS elemental maps presented in Figure 2. In these maps, brighter color intensities correspond to higher concentrations of the respective elements, whereas darker shades indicate lower elemental concentrations. This contrast enables the visualization of compositional gradients and phase segregation across the cross-section of the eutectic samples, providing insight into the spatial evolution of phase development under different crystallization conditions. The EDS maps illustrate the coexistence of two immiscible phases within the eutectic structure, consistent with the formation of YAG and YAP phases. The crystallization rate significantly influences the morphology and scale of these phases. At a low solidification rate of 0.1 mm/min, the system has more time to achieve equilibrium, leading to the formation of larger, more well-defined domains of the two phases. The distinct bright green regions in the Al maps suggest the formation of an Al-rich phase, while the relatively uniform Y distribution with some depletion in the Al-rich regions hints at a Y-rich phase. As the solidification rate increases (0.6 and 0.9 mm/min), the solidification process becomes kinetically limited. This results in a finer, more complex microstructure, where the garnet and perovskite phases interpenetrate on a smaller scale. The diffusion of elements is restricted, leading to a less pronounced segregation and the formation of finer eutectic lamellae/cellular structures. It is noteworthy that at solidification rates up to 0.6 mm/min, the crystal core exhibits a slight enrichment in one of the constituent phases. However, at higher solidification rates, the phase distribution becomes more uniform throughout the microstructure. These findings highlight the role of the crystallization rate in tailoring the microstructure and phase composition of YAG-YAP eutectic systems. By controlling the solidification rate, it is possible to tailor phase domain sizes and enhance the uniformity and separation of constituent phases, which is critical for the development of materials with tailored optical, structural, and thermal properties for targeted applications.

**Figure 2:**
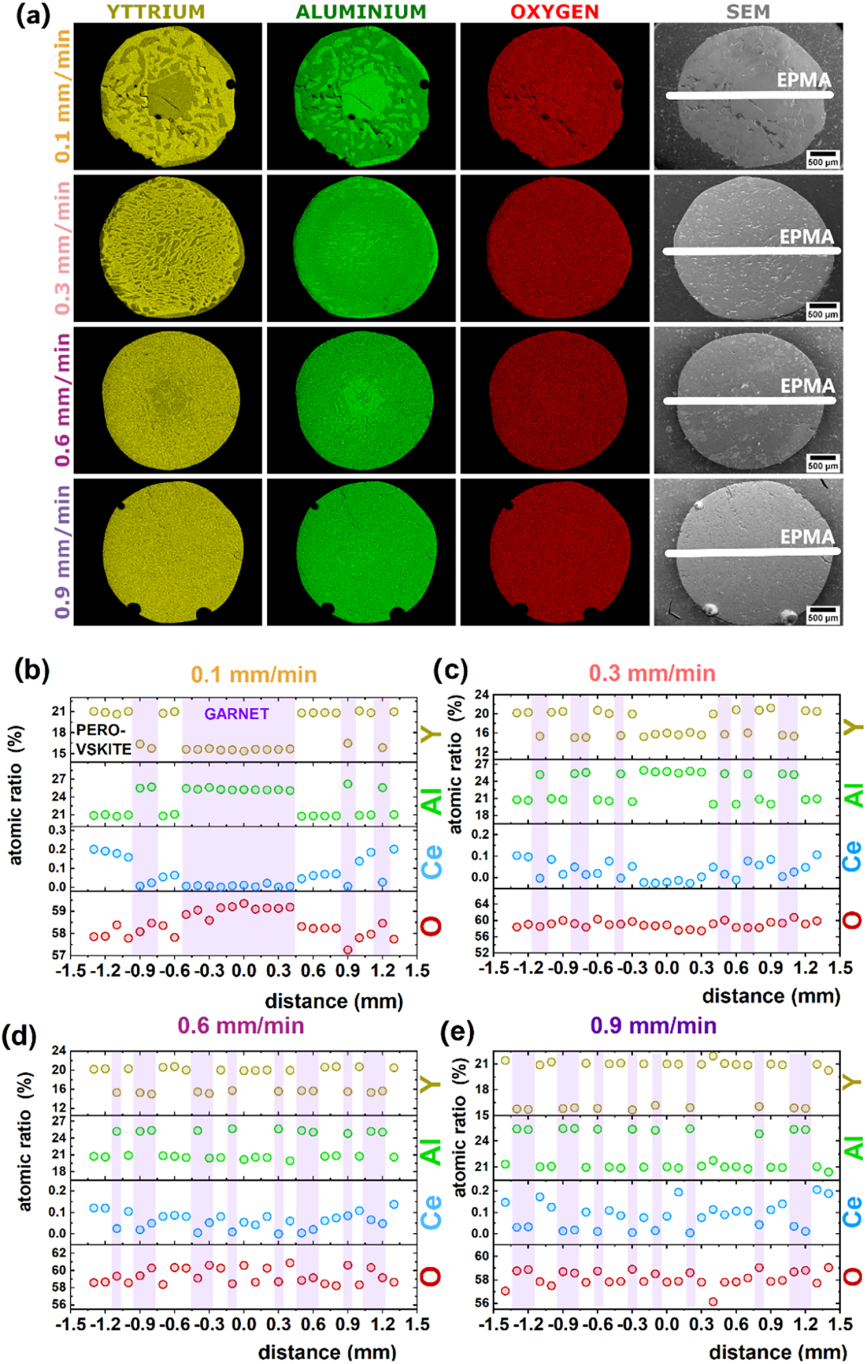
(a) SEM images and corresponding EDS maps for Y (yellow), Al (green), and O (red) elements in Ce^3+^-doped YAG-YAP eutectic crystals solidified at rates 0.1 and 0.9 mm/min. Radial elemental distribution profiles of Y, Al, Ce, and O across the interface of eutectic crystals measured by electron probe microanalysis for samples solidified at speeds of (b) 0.1, (c) 0.3, (d) 0.6, and (e) 0.9 mm/min.

The SEM-EDS elemental maps are insufficient to clearly distinguish between the garnet and perovskite phases due to their similar elemental compositions. To overcome this limitation, optical imaging under blue light illumination (450 nm) was used, taking advantage of the differential luminescence response of the two phases. Specifically, Ce^3+^ ions in the garnet phase absorb blue light and emit yellow luminescence, while the perovskite phase remains transparent under the same conditions.^12,15^ This contrast enables a clear visual differentiation between the two phases. Microimages captured using this method are presented in Figure S1 in the Supporting Information (SI). The micrographs revealed that the core regions of eutectic crystals solidified at slower rates (0.1–0.6 mm/min) exhibit a hypoeutectic structure, dominated by the garnet phase with dispersed inclusions of the perovskite phase. In contrast, samples solidified at higher rates demonstrated the formation of a well-defined, refined eutectic lamellar microstructure, indicative of more complete coupled growth.

Figure 2b–e presents the EPMA point composition analyses for eutectic crystals solidified at rates of 0.1, 0.3, 0.6, and 0.9 mm/min. Quantitative Ce^3+^ concentrations were determined on polished cross sections by using wavelength-dispersive spectroscopy (WDS), with a detection sensitivity better than 100 ppm. Calibration was performed using a CePO_4_ standard, and data were corrected by using the PAP (ϕ−ρ–z) matrix correction method. The resulting phase-resolved chemical compositions are in good agreement with the corresponding EDS elemental maps, thereby confirming the accuracy of phase identification and validating the phase assignment throughout the eutectic microstructures.^23^ At the lowest rate (0.1 mm/min), the eutectic core is predominantly garnet, evidencing the compositional heterogeneity introduced by slow solidification. In this sample, the Ce^3+^ distribution exhibits the well-known rim-enriched/core-depleted pattern characteristic of melt growth when the activator is strongly rejected at the interface.^24–26^ In contrast, eutectics solidified at 0.3 and 0.9 mm/min show significantly improved homogeneous radial distributions of both phases and of Ce^3+^ ions across the cross-section. Table 2 summarizes the phase compositions, showing that Y and Al remain essentially constant with solidification rate, whereas the incorporated Ce^3+^ ions vary most strongly.

**Table 2:** Variation in Ce^3+^ Ion Concentrations within the Garnet and Perovskite Phases as a Function of the Crystallization Rate

	**effective chemical composition**	
**rate of solidification** (mm/min)	**garnet**	**perovskite**
0.1	Y_2.9975_ **Ce**_**0.0025**_Al_5_O_12_	Y_0.974_ **Ce**_**0.026**_AlO_3_
0.3	Y_2.9972_ **Ce**_**0.0028**_Al_5_O_12_	Y_0.975_ **Ce**_**0.025**_AlO_3_
0.6	Y_2.9969_ **Ce**_**0.0031**_Al_5_O_12_	Y_0.975_ **Ce**_**0.025**_AlO_3_
0.9	Y_2.9960_ **Ce**_**0.0040**_Al_5_O_12_	Y_0.977_ **Ce**_**0.023**_AlO_3_

These trends are caused by the different segregation (distribution) coefficients of Ce^3+^ ions in the YAG and YAP phases. In the garnet lattice, the equilibrium segregation coefficient is around *k*_eff_∼0.05–0.1.^27,28^ Under slow solidification conditions, the Burton–Prim–Slichter (BPS) model predicts that the effective segregation coefficient (*k*_eff_) increases with interface velocity due to the reduction in the thickness of the solutal boundary layer.^28,29^ Consequently, the slower solidification rates enhance solute rejection, leading to pronounced Ce^3+^ concentration gradients between the crystal core and the rim. In contrast, higher solidification rates promote more uniform Ce^3+^ incorporation.^28^ By contrast, in the perovskite lattice, the segregation coefficient of Ce^3+^ ions is relatively high (*k*_eff_ ≈ 0.4–0.5),^30^ resulting in a weak dependence of dopant incorporation on the solidification rate.^28^ Consequently, the Ce^3+^ ions concentration in the YAP phase remains nearly constant across the studied solidification rates (Table 2). These differences in Ce^3+^ ion segregation behavior can be attributed to the crystallographic distinctions between the YAG and YAP lattices. In the cubic YAG structure, Y^3+^ ions occupy a dodecahedral YO_8_ coordination site within a rigid corner-sharing AlO_4_/AlO_6_ framework. This site exhibits two distinct Y–O bond lengths of approximately 2.31 and 2.43 Å (four bonds each), forming a relatively size-constrained polyhedral cage.^28^ In contrast, the orthorhombic YAP lattice also features Y^3+^ ions in 12-fold coordination, where the YO_12_ polyhedron in the perovskite structure is more distorted, with Y–O bond lengths ranging from ∼2.24 to 3.26 Å.^31^ This broader bond length distribution reflects a more compliant local environment arising from cooperative octahedral tilting. This structural contrast implies a lower elastic strain energy associated with Ce^3+^ → Y^3+^ substitution in YAP relative to YAG lattice. The increased elastic compatibility in the YAP lattice facilitates more favorable incorporation of Ce^3+^ ions, resulting in a higher effective segregation coefficient, and exhibits negligible sensitivity to the solidification rate. Conversely, the sterically constrained Y-site in YAG lattice corresponds to a significantly low Ce^3+^ segregation coefficient and pronounced dopant concentration gradients that are strongly dependent on the solidification rate.^28^

The segregation behavior of Ce^3+^ ions demonstrates a pronounced dependence on the solidification rate with distinct trends observed between the garnet and perovskite phases. In the garnet phase, the segregation coefficient of Ce^3+^ increases significantly with rising solidification rates, indicating the enhanced incorporation of Ce^3+^ into the solid phase under rapid solidification conditions. Conversely, in the YAP phase, the segregation coefficient of Ce^3+^ exhibits a slight decline as the solidification rate increases. This inverse trend is primarily due to the preferential partitioning of Ce^3+^ into the garnet phase at higher solidification rates, which limits its availability for incorporation into the YAP structure.

The absorption spectra recorded from the core regions of each eutectic crystal provide further confirmation of the microstructural differences associated with varying solidification rates. Specifically, the spectra indicate that eutectic crystals solidified at lower rates (0.1 and 0.6 mm/min) exhibit features consistent with a hypoeutectic structure, dominated by the garnet phase with dispersed perovskite inclusions. In contrast, a crystal solidified at a higher rate (0.9 mm/min) displays spectral characteristics indicative of a well-developed lamellar eutectic structure (Figure S2 of the SI).

### 3.2 Photoconversion and Photoluminescence Characteristics and Temperature Sensitivity Behavior

Figure 3 summarizes the photoconversion and emission dynamics of Ce^3+^-doped YAG-YAP eutectic crystals grown at different solidification rates under blue LED excitation (450 nm). In Figure 3a, the normalized emission spectra show a clear dependence on the growth rate. The sample grown at 0.1 mm/min exhibits a higher intensity in the blue region and a higher correlated color temperature (CCT = 7974 K), resulting in a cold-white emission. As the growth rate increases to 0.3–0.9 mm/min, the intensity in the red-yellow region rises, shifting the CCT toward warm-white light (4623 and 4348 K, respectively). This shift indicates increased scattering and reabsorption of blue photons due to a uniformly developed lamellar structure with comparable YAG and YAP phase dimensions in submicrometer-scale sizes. The luminous efficacy (LE) reaches a maximum of 142 lm/W at 0.6 mm/min, while the color rendering index (CRI) slightly decreases with an increasing growth rate. Notably, an increase in the solidification rate leads to a slight decrease in the relative volume fraction of the garnet phase (Table 1). Concurrently, the concentration of Ce^3+^ ions increases, and the YAG–YAP lamellar microstructure becomes a uniformly developed lamellar structure with comparable YAG and YAP phase dimensions in submicrometer-scale sizes (Figure 1). These morphological and compositional changes result in two key effects: (i) the well-defined lamellar structure with comparable YAG and YAP phase dimensions enhances the scattering of incident blue excitation light, thereby reducing its transmittance through the sample and increasing its absorption by Ce^3+^ ions; and (ii) the elevated Ce^3+^ concentration (Table 1) further contributes to the overall enhancement of Ce^3+^ emission intensity. In contrast, the eutectic crystal solidified at the lowest rate (0.1 mm/min) exhibits a nonuniformly developed lamellar structure, with YAG and YAP phases having dissimilar dimensions on the submicrometer scale. As a result, minimal scattering and maximal transmittance occur, leading to less efficient excitation of Ce^3+^ ions in the YAG phase and correspondingly lower emission intensity.

**Figure 3:**
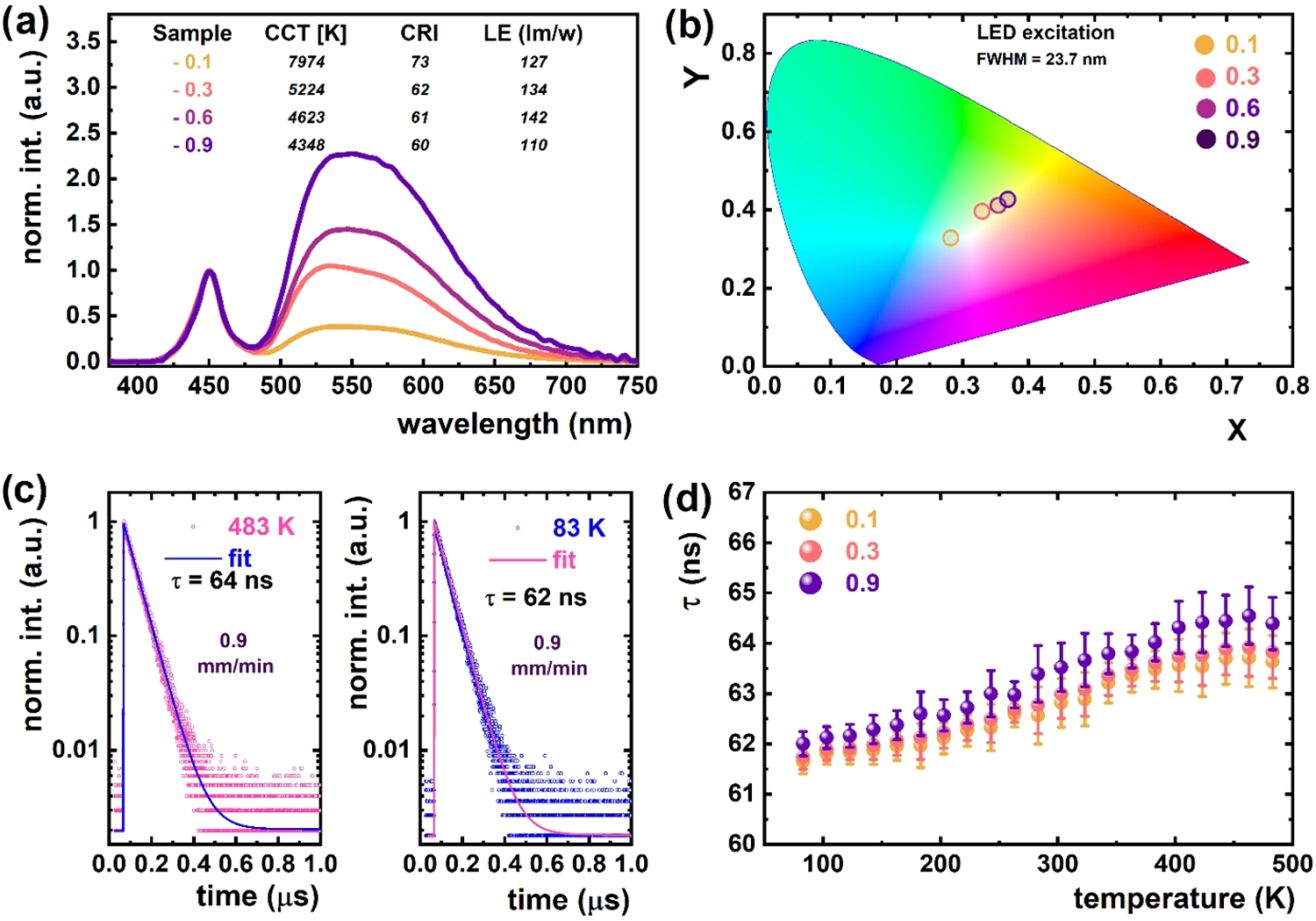
(a) Normalized photoconversion spectra for different solidification rates showing tunable CCT, CRI, and luminous efficacy under 450 nm LED excitation. (b) CIE 1931 chromaticity diagram indicating color coordinates of emissions. (c) Representative decay curves for Ce^3+^ emission (λ_em_ = 570 nm) at 83 and 483 K under 460 nm excitation for crystal solidified at 0.9 mm/min. (d) Temperature dependence of Ce^3+^ decay times (λ_exc_ = 460 nm, λ_emi_ = 570 nm) for samples grown at 0.1 and 0.9 mm/min, showing no quenching up to 483 K.

Figure 3b presents the chromaticity coordinates plotted on the CIE 1931 diagram. The data confirm the tunability of the emission color from cool to warm white by adjusting the solidification rate. The 0.1 mm/min sample lies closer to the daylight region, while the 0.9 mm/min sample shifts toward the natural-white region, consistent with the spectral trends. The differences in photoluminescence behavior, despite the similar phase ratios of Ce^3+^-doped YAG and YAP, can be attributed to variations in the microstructural scale and distribution of the eutectic composite induced by different solidification rates. Specifically, at the lowest solidification rate (0.1 mm/min), the microstructure comprises relatively coarse domains, with YAG and YAP phases reaching submillimeter dimensions. In this case, the YAP phase, which is optically transparent to blue excitation light, permits substantial transmission of incident light through the material with minimal scattering. Consequently, the excitation of Ce^3+^ ions within the YAG phase is less efficient, resulting in reduced yellow emission, while a significant portion of the blue light is transmitted through the eutectic crystal. This leads to a cooler emission color temperature. In contrast, samples solidified at higher rates exhibit a more refined eutectic morphology, namely, a uniformly developed lamellar structure with YAG and YAP domains of comparable size in the submicrometer range. This submicrometer-scale morphology significantly increases the density of interphase boundaries, thereby enhancing the scattering of blue excitation light. The scattered photons have a higher probability of interacting with Ce^3+^ ions in the YAG phase, promoting more effective excitation and partial reabsorption. As a result, the overall transmittance of blue light is reduced, while the yellow emission becomes more pronounced, shifting the correlated color temperature toward the warm-white region.^32^

Figure 3c presents photoluminescence decay profiles of Ce^3+^ emission in the garnet phase, measured at 83 and 483 K for the sample solidified at a rate of 0.9 mm/min. The decay was recorded under 460 nm excitation with the emission monitored at 570 nm. All decay profiles are well-fitted using a single-exponential function, indicating a dominant radiative recombination mechanism. The extracted decay time values (τ = 62 ns at 83 K and τ = 64 ns at 483 K) show minimal variation with temperature. Figure 3d shows the temperature dependence of decay times for Ce^3+^ emission in eutectic crystals solidified at rates of 0.1 and 0.9 mm/min. In both cases, the decay time slightly increases with temperature, indicating the absence of thermal quenching up to 483 K. The observed thermal stability can be attributed to the homogeneous distribution of YAG and YAP phases within the composite matrix, which promotes uniform thermal behavior. Additionally, the comparable thermal conductivities of both phases facilitate efficient heat dissipation, thereby minimizing localized thermal gradients and contributing to the overall stability of the dual-phase system.^33,34^ Additionally, the observed prolongation of the decay time with increasing temperature is associated with reabsorption processes.^35,36^ These are driven by the thermal broadening of both excitation and emission bands of Ce^3+^ ions, which enhances the probability of photon reabsorption and subsequent delayed emission. This effect is more pronounced in the sample grown at 0.9 mm/min, likely due to its slightly higher Ce^3+^ concentration, which increases the likelihood of reabsorption events.^36^ To further confirm the absence of thermal quenching, a long-term thermal aging experiment was conducted under continuous excitation and elevated temperature conditions.^37,38^ As detailed in the SI (Figure S3), the emission intensity was observed to increase slightly over time during sustained irradiation at 450 K. This behavior suggests a self-optimization effect induced by the combined influence of thermal and photonic stress,^12^ further supporting the excellent photothermal stability of these eutectic crystals. The luminescent properties of Ce^3+^-doped eutectics arise from electronic transitions between the 5d excited state and the 4f ground state of Ce^3+^ ions, and have been extensively studied and documented in the literature^39^ with YAG:Ce^3+^ and YAP:Ce^3+^ being the most widely utilized among scintillating materials.^40,41^ The repeatability of the decay time measurements was evaluated by performing three consecutive measurements on a Ce^3+^-doped YAG-YAP eutectic crystal (0.3 mm/min). The results show excellent agreement across the full temperature range, with low variability between replicates; see Figure S4a,b in the SI. The photoluminescence (PL) emission spectra of the Ce^3+^-doped eutectic crystal (0.9 mm/min) under various excitation wavelengths are systematically investigated to identify the optimal conditions for dual-phase excitation. As shown in Figure S5 (SI), excitation at 315 nm yields balanced emission from both the perovskite (YAP) and garnet (YAG) phases, making it the most suitable wavelength for ratiometric luminescence thermometry.

Room-temperature emission spectra of the Ce^3+^-doped YAG-YAP eutectic crystals reveal two distinct bands centered around 375 nm and 550 nm, which correspond to the characteristic Ce^3+^ emissions from YAP:Ce^3+^ and YAG:Ce^3+^, respectively (Figure 4a). To enable the simultaneous observation of both emission bands, photoluminescence spectra were recorded under excitation at λ_exc_ = 315 nm. This wavelength corresponds to the excitation bands of the Ce^3+^ ions present in both the YAG and YAP phases, thereby ensuring effective excitation of both luminescent centers (Figure 4b). The optical excitation at 315 nm is directly resonant with the 4f → 5d_2_ transition of Ce^3+^ ions in the YAG phase and the 4f → 5d_1_ transition in the YAP phase, thereby enabling efficient excitation of both luminescent centers. This underscores the key importance of selecting an appropriate excitation wavelength to facilitate simultaneous observation of dual-phase emission. Under this excitation wavelength, the emission spectra are initially dominated by Ce^3+^ ions in YAP (Figure 4a). However, the luminescence intensity of Ce^3+^ ions in YAG exhibits a slight enhancement with increasing solidification rates. This behavior is primarily attributed to the elevated incorporation of Ce^3+^ ions into the garnet phase, which results from an increase in the effective segregation coefficient during solidification, see Figure 2 and Table 2. As the solidification rate rises within the Ce^3+^-doped YAG-YAP eutectic system, the partitioning of Ce^3+^ ions becomes more favorable, thereby promoting higher dopant concentrations in the YAG phase and leading to stronger photoluminescence emission. Consequently, the relative intensities of the two emission bands shift, resulting in a slight change in the perceived emission color from greenish hues at lower growth rates to more yellow hues as the growth rate increases (Figure 4c).

**Figure 4:**
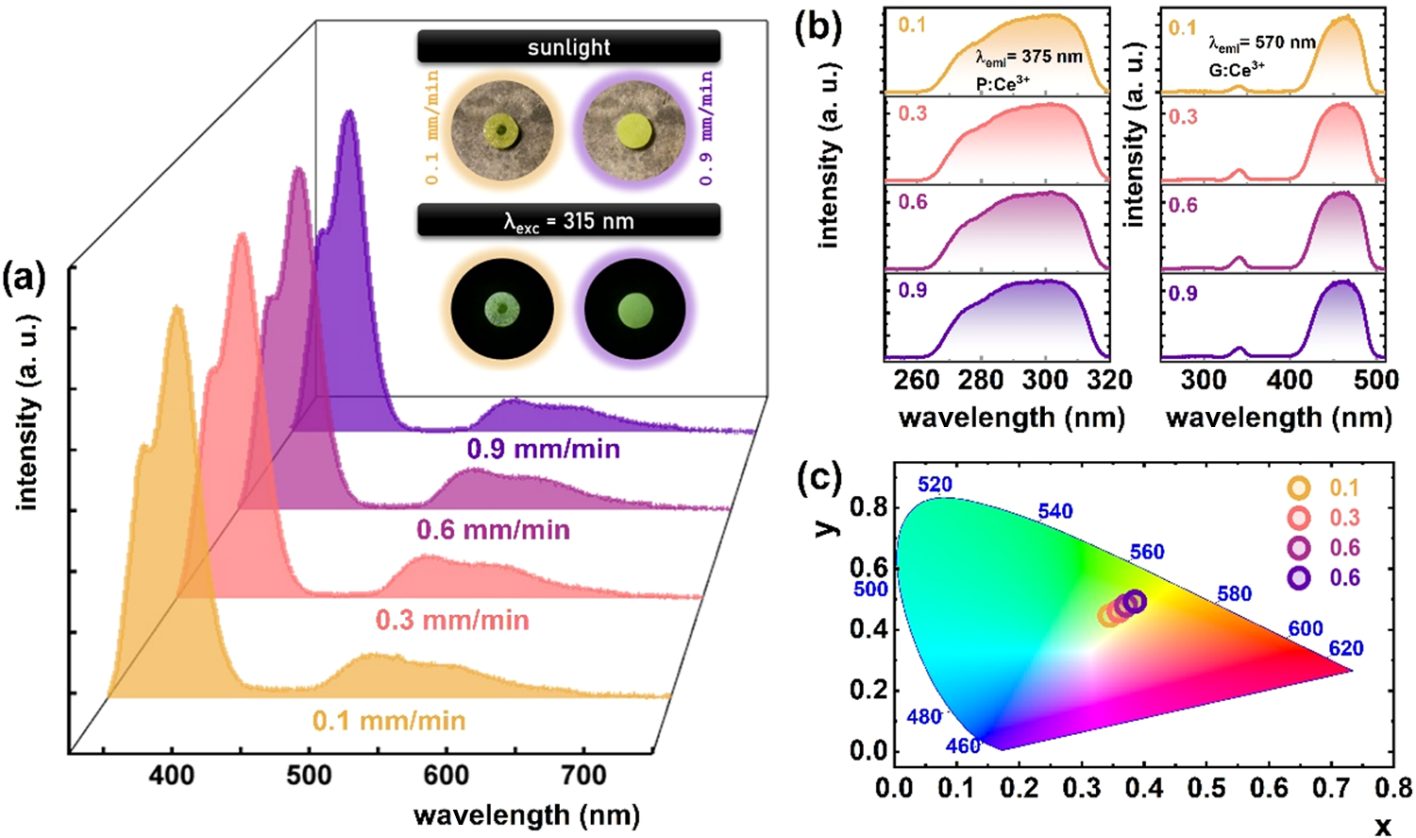
(a) Comparison of normalized emission spectra (λ_exc_ = 315 nm) of Ce^3+^-doped YAG-YAP eutectic crystals obtained with different growth rates, measured at 83 K (inset presents the photographs of samples obtained with 0.1 mm/min (left) and 0.9 mm/min (right) growth rates in sunlight (top) and upon λ_exc_ = 315 nm excitation (bottom)). (b) Corresponding excitation spectra for λ_em_ = 375 nm (dashed line) and λ_em_ = 570 nm (solid line). (c) The CIE 1931 chromatic coordinates of the light emitted by these samples at 83 K.

The luminescence spectra of Ce^3+^-doped YAG-YAP eutectic crystals, measured as a function of temperature over the 83–483 K range, demonstrate that the emission intensity of Ce^3+^ in both YAP and YAG increases progressively with rising temperature, see Figure 5a (0.6 mm/min). Although this behavior deviates from the typical thermal quenching observed in most luminescent materials, it is explained by a thermally stimulated luminescence (TL) mechanism mediated by Ce^3+^ ions in the YAG phase.^42^ Under 315 nm excitation, the 4f → 5d_2_ electronic transition of Ce^3+^ ions is activated within the garnet structure. The energy barrier between the conduction band minimum (CBM) and the 5d_2_ excited state of Ce^3+^ in YAG is relatively low, which facilitates thermal ionization of the excited electrons into the conduction band at slightly higher temperatures.^43^ Subsequently, the released electrons are captured by defect-related trapping centers within the host matrix. As the temperature increases, these trapped electrons are thermally released and subsequently recombine with Ce^3+^ ions in both the YAG and YAP phases. This thermally activated recombination process leads to an enhancement of the emission intensity rather than quenching, consistent with the observed luminescence behavior.^42,43^ Furthermore, as the temperature increases, the Ce^3+^ ion emission maxima of both the perovskite and garnet phases progressively shift toward lower energies (redshift). This behavior is attributed to the thermal broadening of both excitation and emission bands, which becomes more pronounced at elevated temperatures and leads to a gradual decrease in the emitted photon energy.^12,36^ This spectral broadening facilitates the reabsorption of photons corresponding to the 5d_1_ → ^2^F_5/2_ transition by neighboring Ce^3+^ ions. Additionally, nonradiative energy transfer occurs to perturbed Ce^3+^ ions, which subsequently emit at slightly lower energies. As a result, the 5d_1_ → ^2^F_7/2_ transition becomes increasingly dominant, contributing more significantly to the overall luminescence intensity (i.e., the combined 5d_1_ → ^2^F_5/2_ + ^2^F_7/2_ emissions). This leads to a continuous redshift in the emission-band maximum with rising temperature.^12,36,44^ This result is consistent with the observed reabsorption effect, which contributes to the prolongation of the Ce^3+^ decay times, as shown in Figure 3d. Due to instrumental constraints, a comparable kinetic analysis of Ce^3+^ ions in the YAP phase could not be conducted. Notably, the thermally induced redshift in the luminescence of Ce^3+^-doped YAG is slightly more pronounced than that observed in Ce^3+^-doped YAP. This differential behavior leads to a subtle but measurable temperature-dependent variation in the emission color, as evidenced by the shift in chromaticity coordinates within the CIE 1931 diagram (Figure 5b). A detailed analysis of the integrated luminescence intensity as a function of temperature reveals that, for Ce^3+^-doped YAG, the emission intensity increases up to a 5-fold between 83 and 400 K at the solidification rate of 0.9 mm/min, see Figure 5c,d. For Ce^3+^-doped YAP, the intensity increases in the same temperature range are somewhat smaller, ranging to 3-fold (at 0.9 mm/min). The lower thermal enhancement observed in Ce^3+^-doped YAP is likely due to phonon-assisted energy transfer to Ce^3+^-doped YAG.^14,45^ Given the energy mismatch between the fundamental and excited levels in the two phases, such a transfer becomes more probable at elevated temperatures due to increased phonon activity and bands broadening. Although a thermal broadening of the Ce^3+^ excitation and emission bands is observed for all samples, a more significant decrease in excitation band intensity is noted at lower solidification rates compared to higher ones. The reduction in excitation band intensity with increasing temperature is attributed to the thermal population of higher vibrational states (ν > 0), which leads to a simultaneous broadening of the bands and a decline in their intensity.^36,39^ This phenomenon arises from enhanced electron–phonon coupling at elevated temperatures, which redistributes the oscillator strength over a broader spectral range. The markedly stronger decrease in excitation band intensity observed in the eutectic sample solidified at a rate of 0.1 mm/min, compared to the sample solidified at 0.9 mm/min, is primarily due to the significantly lower concentration of Ce^3+^ ions in the former. This correlation is evident from the compositional data presented in Table 2 and Figure 2. The different thermal behaviors of the Ce^3+^-doped YAP and Ce^3+^-doped YAG emission bands allow for the development of a ratiometric luminescence thermometer. In this system, the luminescence intensity ratio (LIR) between the two bands serves as the thermometric parameter:

2 $$LIR=\frac{\int_{510\;nm}^{520\;nm}I\left(Y{AG:Ce}^{3+}\right)d\lambda }{\int_{343\;nm}^{353\;nm}I\left({YAP:Ce}^{3+}\right)d\lambda }$$

As shown in Figure 5e, the LIR exhibits a similar temperature dependence across all growth rates: a slight decrease in LIR is observed from 83 K to approximately 103 K, followed by a monotonic increase up to 400 K, beyond which LIR begins to decline. Since ratiometric luminescence thermometers require a monotonic response within the operational temperature range, the effective working range of this system is limited to approximately 103–400 K. To quantify the thermal response, the relative sensitivity (*S*_R_) was calculated using the standard formula:

3 $$S_{R}=\frac{1}{LIR}\frac{\Delta LIR}{\Delta T}\times 100\%$$

As illustrated in Figure 5f, *S*_R_ exhibits two distinct maxima: one around 180 K and another near 300 K. The highest *S*_R_ value, 0.47% K^–1^, was recorded at 180 K for a growth rate of 0.9 (Figure 5f). Lower growth rates result in a slight decrease in the relative sensitivity values. The repeatability of the temperature-dependent luminescence measurements was assessed by performing three consecutive measurements on the same Ce^3+^-doped YAG–YAP eutectic crystal, solidified at a rate of 0.3 mm/min, under identical conditions. The results showed excellent agreement across all temperature points with low standard deviations and coefficients of variation below 0.3%, confirming high measurement precision. A detailed analysis, including individual replicate curves and error quantification, is provided in Figure S6 in the SI.

**Figure 5:**
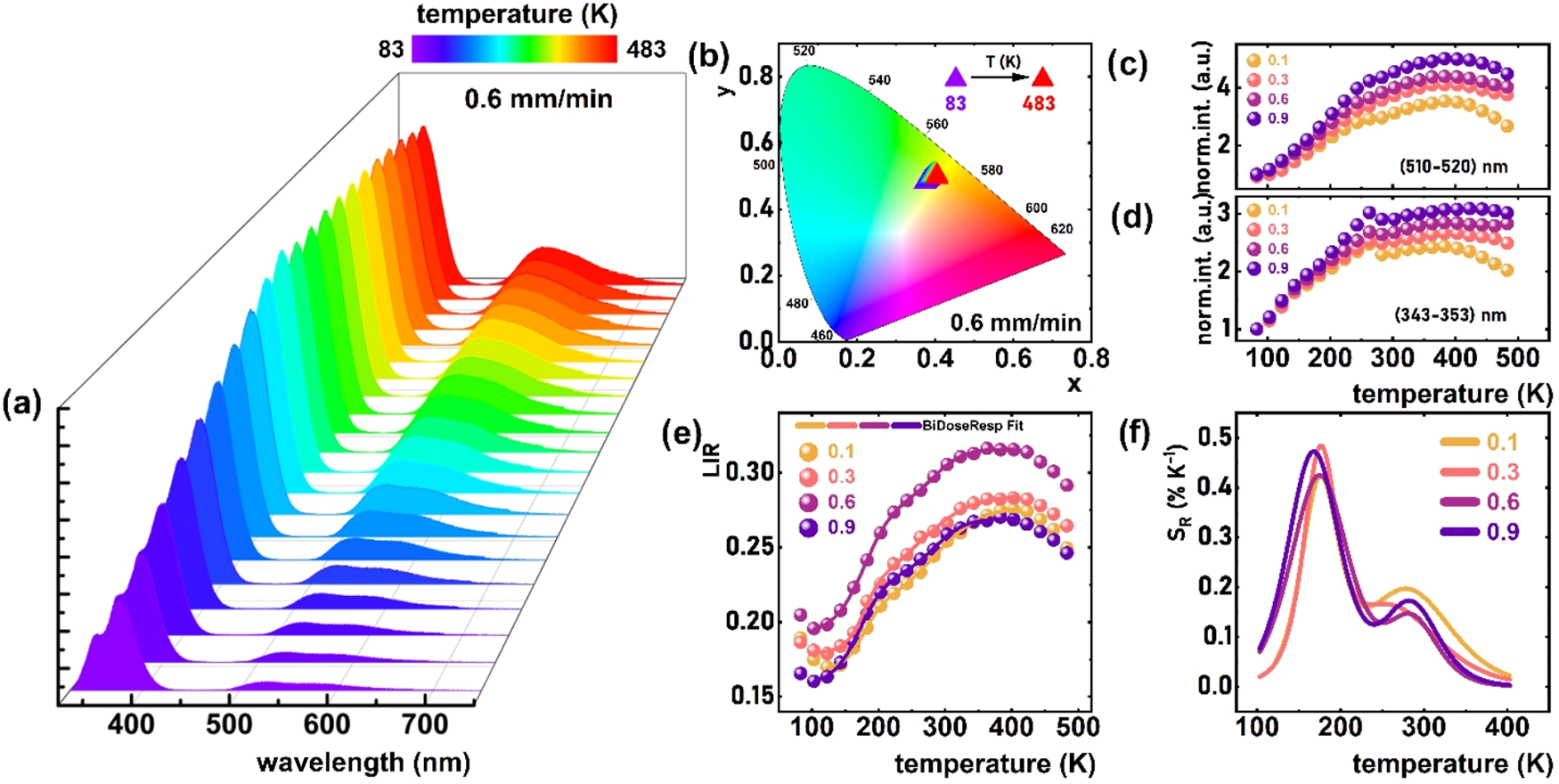
(a) Thermal dependence of emission spectra of the Ce^3+^-doped YAG-YAP eutectic crystal (0.6 mm/min) under λ_exc_ = 315 nm and (b) corresponding influence of temperature on the CIE 1931 chromatic coordinates (0.6 mm/min). (c) The influence of temperature on the emission intensities integrated into 510–520 nm and (d) 343–353 nm. (e) Spectral ranges for different growth rates of Ce^3+^-doped YAG-YAP eutectic crystals; thermal dependence of LIR values and (f) the corresponding *S*_R_ for Ce^3+^-doped YAG-YAP eutectic crystals obtained with different growth rates.

### 3.3 X-ray Luminescence Characteristics and Temperature Sensitivity Behavior

Analysis of the luminescence spectra of Ce^3+^-doped YAG-YAP eutectic crystals under X-ray excitation reveals that the spectral positions of the Ce^3+^ emission bands in both YAG and YAP are comparable to those observed under photoluminescence excitation (Figures 6a and S7–S9 in SI). However, the relative intensity of the emission band in Ce^3+^-doped YAG is significantly higher compared to the Ce^3+^-doped YAP band under X-ray excitation than it is under λ_exc_ = 315 nm photonic excitation. This discrepancy is attributed to the distinct excitation mechanisms governing the Ce^3+^ luminescence under X-ray excitation. In X-ray luminescence, Ce^3+^ emission occurs via a scintillation process, wherein high-energy radiation generates electron–hole pairs that migrate through the electronic structure of the host material until they are captured by Ce^3+^ luminescent centers in both the YAG and YAP phases. During this migration, charge carriers may be trapped by various defect centers, such as *Y*_Al_^*x*^, Lu_Al_^*x*^ dislocations, and oxygen vacancies^46–48^ as well as trace impurities,^49–52^ see Figures S7–S9 in SI. As the temperature increases, these trapped charge carriers are thermally released and subsequently recaptured by Ce^3+^ ions in both garnet and perovskite phases, thereby enhancing Ce^3+^ emission.^24,53^ Additionally, at elevated temperatures, the trap depths decrease, rendering them less effective at capturing electrons, as the available thermal energy surpasses the trap energy barriers. As a result, the excitation energy is more efficiently transferred to the Ce^3+^ ions, leading to a significant increase in emission intensity. A similar thermally induced enhancement of luminescence intensity in various Ce^3+^-based scintillating materials has been reported previously and is explained by thermally activated energy transfer from defect traps to the 5d excited states of Ce^3+^ ions.^24,54^ Nevertheless, the Ce^3+^ emission from the YAP phase remains dominant, as it is consistent with the photoluminescence spectra presented in Figure 5. This predominance is attributed to the lower concentration of Ce^3+^ ions in the YAG phase relative to that in the YAP phase, which reduces the likelihood of radiative recombination within the YAG matrix and thereby accentuates the disparity in emission intensity. Furthermore, the higher density of YAP:Ce (5.37 g/cm^3^) relative to YAG:Ce (4.57 g/cm^3^) enhances the X-ray stopping power of the YAP phase, leading to more efficient absorption of incident X-rays and, consequently, stronger luminescence from Ce^3+^ in YAP than in YAG.^55^ Such distinctions in the thermal response of luminescence can be effectively used in luminescent thermometry. As shown in Figure 6b, an increase in temperature results in an increase in the luminescence intensity ratio up to approximately 410 K for all analyzed samples. Beyond this temperature point, LIR values decrease until around 450 K, above which a further increase is observed. This observed temperature-dependent behavior determined thermally usable range of scintillating thermometer from 325 K up to 410 K and led to elevated *S*_R_ values, peaking around 325 K with a maximum *S*_R_ of 0.7% K^–1^ for a growth rate of 0.6 mm/min. A second *S*_R_ maximum is observed near 395 K, with an *S*_R_ of approximately 1.1% K^–1^ for a sample with a growth rate of 0.6 mm/min. While the general trend of the thermal LIR response is consistent across the studied samples, minor variations in *S*_R_ values are evident, attributable to differences in the initial LIR values.

**Figure 6:**
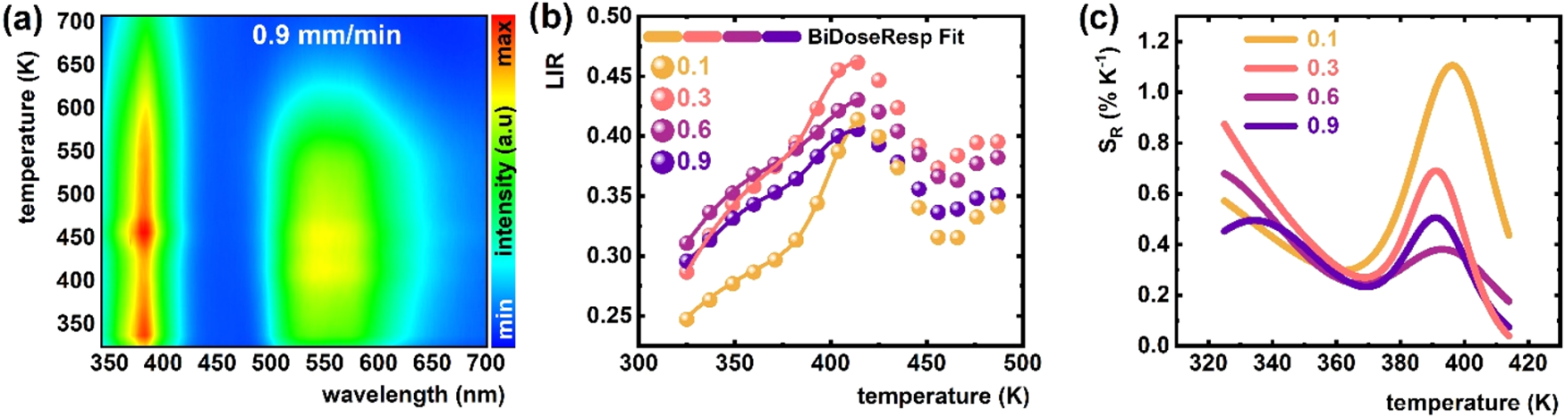
(a) Luminescence thermal map of Ce^3+^-doped YAG-YAP eutectic crystal (0.9 mm/min) under X-ray excitation. (b) Thermal dependence of LIR and (c) the corresponding *S*_R_ for Ce^3+^-doped YAG-YAP eutectic crystals obtained with different growth rates.

To gain further insight into the role of trapping centers and their influence on Ce^3+^ luminescence in doped YAG–YAP eutectic crystals, the defect-related luminescence was additionally investigated in an undoped YAG–YAP eutectic crystal solidified at a rate of 0.7 mm/min, see Figure S10. Under 315 nm excitation, the undoped sample exhibits a broad ultraviolet emission band between 320 and 420 nm, which is attributed to radiative recombination at intrinsic point defects, such as oxygen vacancies and antisite defects, introduced during the high-temperature growth process. This emission demonstrates strong thermal quenching, as increasing temperature promotes carrier detrapping and enhances nonradiative recombination through phonon-assisted processes. In contrast, Ce^3+^-doped YAG–YAP eutectic crystals show a thermally enhanced Ce^3+^ emission under the same excitation conditions. This divergent behavior is explained by the dual function of defects; while they act as luminescent centers in the undoped material, they serve as intermediate trap states in the doped system, from which thermally released carriers are transferred to Ce^3+^ ions. As the temperature increases, this trap-mediated energy transfer becomes more efficient, enhancing the radiative output of the Ce^3+^ centers. The comparison reveals that the presence of Ce^3+^ fundamentally alters the role of defect states, from competing recombination centers to sensitizing Ce^3+^ luminescence.

## 4 Conclusions

Ce^3+^-doped YAG-YAP eutectic crystals were successfully synthesized via directional solidification, enabling precise control over the microstructure and dopant segregation by varying the growth rate from 0.1 to 0.9 mm/min. The resulting lamellar microstructure, comprising alternating garnet (YAG) and perovskite (YAP) phases, exhibited rate-dependent domain refinement and preferential Ce^3+^ incorporation into the garnet matrix at higher solidification rates. Morphological control directly influenced optical behavior: at lower solidification rates, the larger YAP domains allowed high blue light transmittance, whereas higher rates produced finer structures that enhanced blue light scattering and absorption. This structural tunability enabled effective modulation of the correlated color temperature (CCT), spanning from cool to warm white emissions, thereby tailoring the material’s photoconversion characteristics for potential solid-state lighting applications. The Ce^3+^ luminescence in these eutectic crystals exhibited negligible thermal quenching up to 480 K. This exceptional thermal stability is attributed to the uniform distribution of YAG and YAP domains, both of which possess inherently high thermal conductivity. The well-integrated dual-phase network promotes efficient heat dissipation and suppresses localized thermal accumulation, thereby stabilizing the emission performance at elevated temperatures. This feature positions YAG-YAP eutectic composites as promising phosphors for high-power white laser diodes where thermal robustness and emission stability are critical for device reliability and efficiency.

These eutectics demonstrated multimodal luminescence thermometry performance under both photoluminescence (PL) and X-ray-induced scintillation excitation. While PL excitation yielded a maximum relative sensitivity of 0.47% K^–1^ over the 103–400 K range, scintillation-based excitation extended the operational window to 325–410 K and reached a peak sensitivity of 1.1% K^–1^. The undoped YAG–YAP eutectic crystal exhibits broad UV emission arising from intrinsic defects such as oxygen vacancies and antisite substitutions, which act as radiative centers and undergo thermal quenching due to carrier detrapping. In contrast, Ce^3+^-doped YAG–YAP shows enhanced luminescence with increasing temperature, driven by the thermally activated transfer of carriers from shallow traps to Ce^3+^ ions. Photoluminescence decay analysis supports this distinction, with multiexponential decay in the undoped crystal and single-exponential decay in the Ce^3+^ doped sample, characteristic of Ce^3+^ emission in YAP phase.

The observed differences underscore the role of the excitation mode in governing energy transfer and charge carrier dynamics within the dual-phase structure. Furthermore, the ability to perform contactless, self-activated temperature sensing under ionizing radiation without external optical excitation positions these eutectic composites as strong candidates for integration into advanced thermal diagnostic platforms operating in extreme environments such as nuclear reactors, aerospace systems, and high-energy physics detectors. The combination of excitation-selective thermometric response, structural tunability, and robust performance across diverse regimes highlights the versatility of Ce^3+^-doped YAG–YAP eutectic crystals. Moreover, the ability to engineer their optical characteristics through controlled solidification rates offers a pathway toward multifunctional phosphors suitable for both temperature sensing and tunable lighting applications.

## Supplementary Material



## Notes

Crystal phase and morphology; reflected-light (epi-brightfield) plan-view micrographs at 0.1 and 0.9 mm/min (Figure S1); Absorption characteristics; core-region absorption spectra; Ce^3+^ 4f → 5d bands; scattering vs growth rate (Figure S2); photoluminescence characteristics; 450 K continuous excitation aging and measurement-only control (spectra and integrated intensity) (Figure S3); photoluminescence decay kinetics; Ce^3+^ decay times vs temperature, triplicate series with mean ± SD (Figure S4); excitation-dependent photoluminescence; emission at 83 K under 300–330 nm excitation (Figure S5); optimal dual-site excitation at 315 nm; photoluminescence intensity repeatability; temperature-dependent emission (YAG 510–520 nm; YAP 343–353 nm), triplicate with SD (Figure S6); X-ray-excited luminescence and low-T TL; XEL at 20 K; TL glow curves 20–300 K (Figure S7); X-ray-excited luminescence and high-T TL (Figure S7); XEL at 325 K; TL glow curves 300–714 K (Figure S8); integrated TL; TL integrals 50–280 and 300–650 K vs solidification rate (Figure S9); undoped eutectic characterization; photograph; XRD/Rietveld phase fractions (37% YAG–63% YAP); temperature-dependent PL; RT PL vs Ce^3+^-doped; PL decays (Figure S10) (PDF)
