# Supplementary material for: Scintillating and Photoluminescent Ratiometric and Visual Luminescence Thermometry Based on the Ce3+-Doped Eutectic Structures
Source: ACS Appl Mater Interfaces. 2025 Oct 15;17(43):59611–24. doi: 10.1021/acsami.5c16426 (PMC12581115; doi:10.1021/acsami.5c16426)
Supplement: Supplementary file 1 [file am5c16426_si_001.pdf]

SUPPORTING INFORMATION

Scintillating and photoluminescent ratiometric and visual luminescence thermometry based on the Ce^{3+} doped eutectic structures

Karol Bartosiewicz[1*], Maja Szymczak[2], Masao Yoshino[3], Takahiko Horiai[4], Robert Tomala[2,5], Justyna Zeler[6], Aleksandra Owczarek[6], Damian Szymanski[2], Marcin E. Witkowski[7], Vítězslav Jarý[1], Winicjusz Drozdowski[7], Eugeniusz Zych[6], Akira Yoshikawa[3,8], Łukasz Marciniak[2],

[1] Institute of Physics, Czech Academy of Sciences, Na Slovance 1999/2, Praha 18200, Czechia,
[2] Institute of Low Temperature and Structure Research, Polish Academy of Sciences, Okólna 2, Wrocław 50422, Poland,
[3] New Industry Creation Hatchery Center, Tohoku University, 2-1-1 Katahira Aoba-ku, Sendai, Miyagi 980-8577, Japan,
[4] National Institute of Advanced Industrial Science and Technology (AIST), Core Electronics Technology Research Institute, AIST Tsukuba Central 5, 1-1-1 Higashi, Tsukuba, Ibaraki 305-8565, Japan,
[5]Faculty of Chemistry and Geosciences, Vilnius University, Naugarduko g. 24, 03225 Vilnius, Lithuania
[6] University of Wrocław, Faculty of Chemistry, 14 F. Joliot-Curie Street, Wrocław 50383, Poland,
[7] Institute of Physics, Faculty of Physics, Astronomy and Informatics, Nicolaus Copernicus University in Toruń, Grudziądzka 5, Toruń 87100, Poland,
[8] Institute for Materials Research, Tohoku University, 2-1-1 Katahira Aoba-ku, Sendai, Miyagi 980-8577, Japan
Corresponding author's email address: bartosiewicz@fzu.cz

1. Crystal Phase and Morphology

Figure S1 shows surface (plan-view) micrographs of eutectic crystals solidified at 0.1 and 0.9 mm/min, recorded in reflected-light (epi-brightfield). This is not a fluorescence image; the detector records primarily reflected/scattered blue light, and any yellow Ce^{3+} emission is neither spectrally isolated nor enhanced. At 0.9 mm/min, the Ce^{3+}-doped YAG–YAP eutectic forms sub-micrometer lamellae with a high density of phase boundaries. The short optical path within individual Ce^{3+}-doped YAG lamellae limits single-pass absorption of 440–470 nm light, while abundant interfaces increase backscattering of blue light. In epi-brightfield mode, the image gives a greenish appearance. By contrast, in the 0.1 mm/min sample the core is a contiguous, sub-millimeter Ce^{3+}-doped YAG domain with only sparse Ce^{3+}-doped YAP inclusions; scattering is minimal and the blue illumination experiences a long path in garnet phase where it is efficiently absorbed, hence, the core appears distinctly yellow. At the rim of the 0.1 mm/min sample, the microstructure transitions to a YAG-YAP lamellar eutectic with sub-micrometer periodicity comparable to that at 0.9 mm/min. The elevated interface density enhances backscattering of the blue illumination, so in epi-brightfield the rim shows the same grayish cast as the 0.9 mm/min eutectic. The sample solidified at 0.1 mm/min exhibits large, well-separated domains of both YAG and YAP phases, indicative of a hypoeutectic structure. In contrast, the crystal grown at 0.9 mm/min displays a fine lamellar microstructure characteristic of eutectic solidification. A magnified view of the core region in the 0.1 mm/min sample reveals a dominant yellow emission with interspersed transparent regions. The yellow luminescence originates from Ce^{3+} ions within the garnet phase [1, 2], while the transparent areas correspond to the perovskite phase [3]. This suggests that the core of the crystal predominantly comprises the garnet phase with minor inclusions of the perovskite phase, forming a hypoeutectic structure. In contrast, the outer shell displays a more refined eutectic morphology, indicating a spatial variation in the solidification dynamics across the crystal cross-section. A magnified view of the core region in the eutectic crystal solidified at 0.9 mm/min reveals a well-developed lamellar structure, characterized by alternating greenish and grayish tints. The greenish tint, attributed to Ce^{3+} ions in the YAG phase, and the grayish tint,corresponding to the YAP phase, confirm the formation of a refined eutectic microstructure [3, 4].

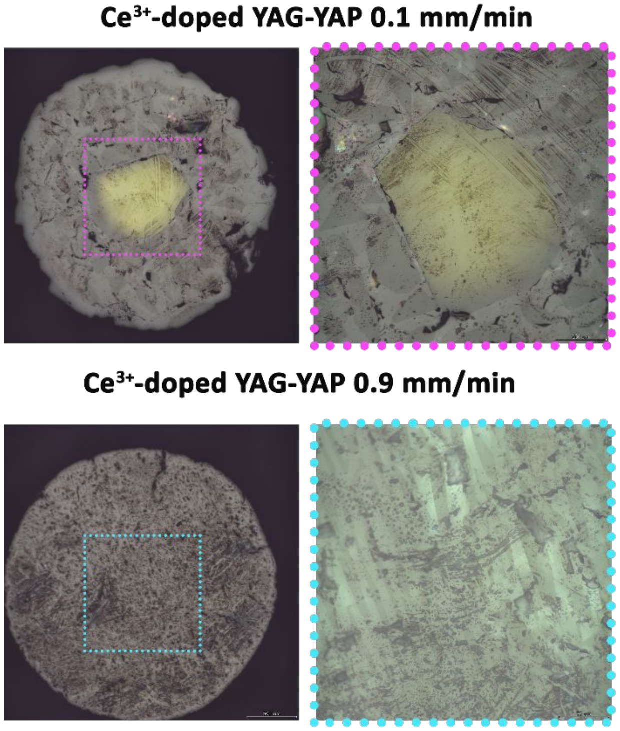

Figure S1. Surface (plan-view) micrographs of eutectic crystals solidified at 0.1 and 0.9 mm/min, recorded in reflected-light (epi-brightfield) under blue illumination (λ = 440 - 470 nm).

2. *Absorption spectra*

Figure S2 presents the absorption spectra of Ce^{3+}-doped YAG-YAP eutectic crystals, recorded from the core regions of each sample. The spectra exhibit characteristic absorption bands centered at approximately 460 nm and 340 nm, corresponding to the $4f \rightarrow 5d_1$ and $4f \rightarrow 5d_2$ electronic transitions of Ce^{3+} ions in the YAG phase [4]. Additionally, a distinct absorption feature near 270 nm is attributed to the $4f \rightarrow 5d_1$ transition of Ce^{3+} ions in the YAP phase [3]. As the solidification rate increases, a noticeable rise in spectral background is observed, accompanied by a decrease in absorption intensity. This behavior is attributed to enhanced light scattering at the interfaces between the finely distributed phases, particularly in samples with well-developed lamellar eutectic structures. This effect is most pronounced in the sample solidified at 0.9 mm/min, which exhibits the most refined eutectic morphology. In contrast, eutectic crystals solidified at slower rates (0.1–0.6 mm/min) show more intense absorption bands associated with Ce^{3+} in the garnet

phase, consistent with the hypoeutectic nature of their cores, where the garnet phase is dominant with fewer interfacial boundaries to induce scattering. As the solidification rate increases, the extent of the hypoeutectic core region progressively decreases. At solidification rates exceeding 0.6 mm/min, a well-developed lamellar eutectic microstructure is fully established, indicating a transition from phase-separated growth to coupled eutectic solidification.

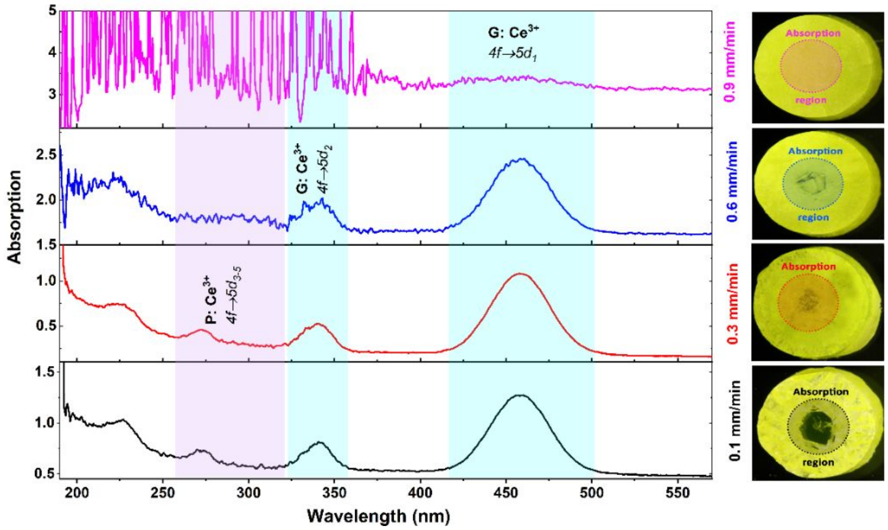

Figure S2. Absorption spectra of Ce^{3+}-doped YAG-YAP eutectic crystals measured at the core region of each sample.

3. *Photoluminescence characteristics*

The photoluminescence (PL) spectra collected during the thermal aging experiment (Figure S3a) exhibit a slight linear increase in emission intensity over a 480-minute period at 450 K under continuous excitation at 460 nm. This trend is corroborated by the temporal evolution of the integrated PL intensity within the 480–720 nm range, shown in Figure S3b, which reveals a steady enhancement over time. These results confirm the absence of thermal quenching and suggest a time-dependent improvement in radiative emission efficiency under sustained thermal and optical excitation. These results are consistent with the thermoluminescence characteristics observed in this eutectic crystals. A similar enhancement in emission intensity at elevated temperatures was recently reported for nonstoichiometric $Y_{2.955}Ce_{0.03}Nd_{0.0135}Al_5O_{12}$ ceramics[4]. The progressive

enhancement in PL intensity is attributed to a combination of thermally and optically activated processes occurring within the material.

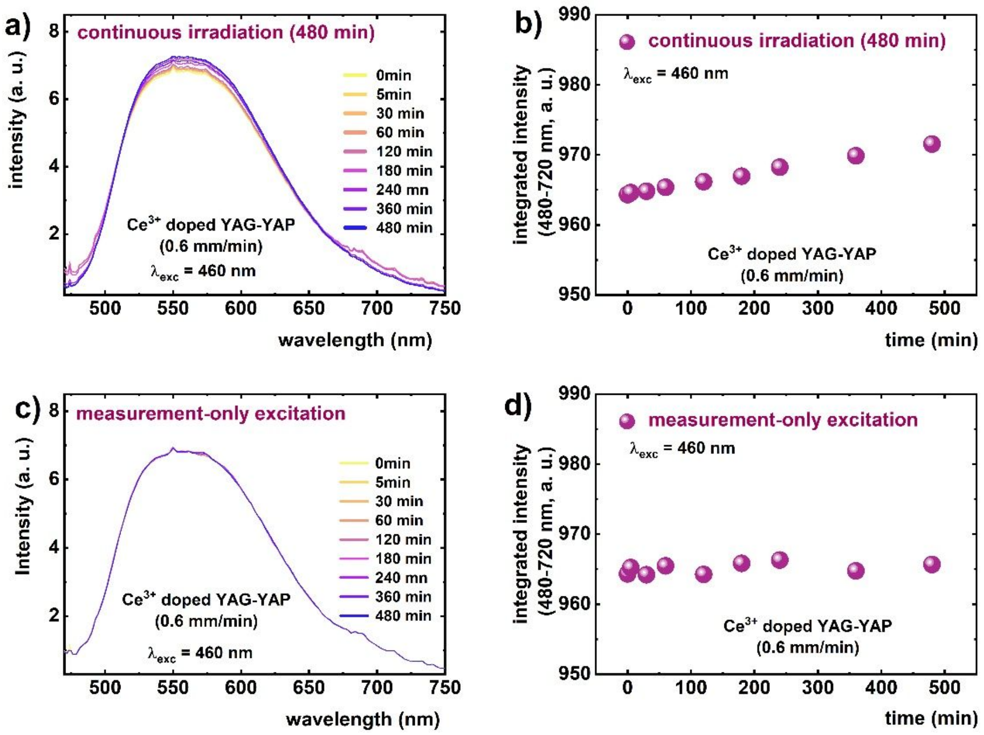

Figure S3. (a) Photoluminescence (PL) spectra of the 0.6 mm/min eutectic crystal recorded at 450 K under continuous irradiation at an excitation wavelength of 460 nm. The spectra were collected at different time intervals ranging from 0 to 480 minutes; (b) Integrated PL intensity (480–720 nm). (c) Photoluminescence spectra acquired under identical thermal and excitation conditions (450 K, λ_{exc} = 460 nm), but where the crystal was exposed to excitation only during the measurement intervals. (d) Integrated PL intensity (480–720 nm) for the measurement-only case over 480 minutes.

Prolonged exposure to elevated temperature facilitates the migration and recombination of intrinsic point defects, such as oxygen vacancies within the host matrix. This defect relaxation and annealing process reduces the density of non-radiative recombination centers, thereby enhancing the probability of radiative transitions. Simultaneously, continuous photoexcitation may promote the dynamic passivation of localized trap states. As these states become progressively neutralized through photon-mediated processes, non-radiative recombination pathways are increasingly suppressed, resulting in the enhancement in PL efficiency. In addition, deep carrier traps are likely to play a significant role during the initial stages of excitation, where a substantial fraction of photo-generated carriers may be temporarily immobilized. As irradiation proceeds and these traps reach saturation, a greater proportion of carriers contributes to radiative recombination, further enhancing the emission intensity. This redistribution of carrier dynamics, combined with possible

thermal activation of trap states and phonon-assisted energy transfer, contributes to the observed time-dependent luminescence enhancement. The long-term thermal aging experiment demonstrates that the material undergoes a form of self-optimization under combined thermal and continuous photoexcitation stress. This behavior underscores the excellent thermal stability of the material and highlights its potential for use in applications demanding long-term operational reliability at elevated temperatures, such as high-power light-emitting devices and scintillators.

Figure S3c and S3d provide a control study to isolate the influence of thermal aging alone, decoupled from the effects of continuous optical excitation. In this experiment, the same Ce^{3+}-doped eutectic crystal was kept at 450 K for 480 minutes, but excitation at 460 nm was applied only during each measurement. The PL spectra shown in Figure S3c exhibit no significant changes in shape or amplitude across the entire duration. Correspondingly, the integrated PL intensity plotted in Figure S3d remains essentially constant. This control experiment confirms that the increase in PL intensity observed under continuous irradiation (Figure S3a–b) is not caused by thermal exposure alone, but rather arises from photoinduced and thermally assisted processes that occur only when the material is continuously excited. The lack of intensity enhancement in the measurement-only condition further supports the conclusion that photon-assisted defect dynamics, such as the filling of traps and redistribution of carriers, are critical to the observed luminescence evolution under prolonged irradiation. These results strongly indicate that thermal annealing alone is insufficient to activate the emission enhancement mechanism. Instead, the synergy between elevated temperature and sustained photoexcitation is necessary to facilitate the progressive reduction of non-radiative pathways and trap saturation effects. This finding revelad that this eutetci is a The long-term thermal aging experiment demonstrates that the material undergoes a form of self-optimization under combined thermal and continuous photoexcitation stress.

To assess the reproducibility, Ce^{3+}-doped YAG-YAP eutectic crystal (0.3 mm/min) was evaluated using three independently prepared batches, measured under identical experimental conditions. At each temperature point T, the corresponding decay times from the three parallel experiments denoted x_1,x_2,x_3 were summarized as s mean ± standard deviation (SD; n=3). The SD was calculated using the unbiased sample estimator (Equation S1):

$$s = \sqrt{\frac{\sum_{i=1}^{n}\left(x_i - \bar{x}\right)^2}{nia}}, where\ ,\bar{x} = \frac{(x_1 + x_2 + x_3)}{n} \qquad \text{(S1)}$$

where x_i - decay time from the *i-th* independently prepared batch (*i=1,2,3*) *n* - number of parallel measurements (n=3), $\bar{x}$- arithmetic mean of the three decay times, and *s* sample standard deviation (SD), reflecting the spread of values across independent batches.

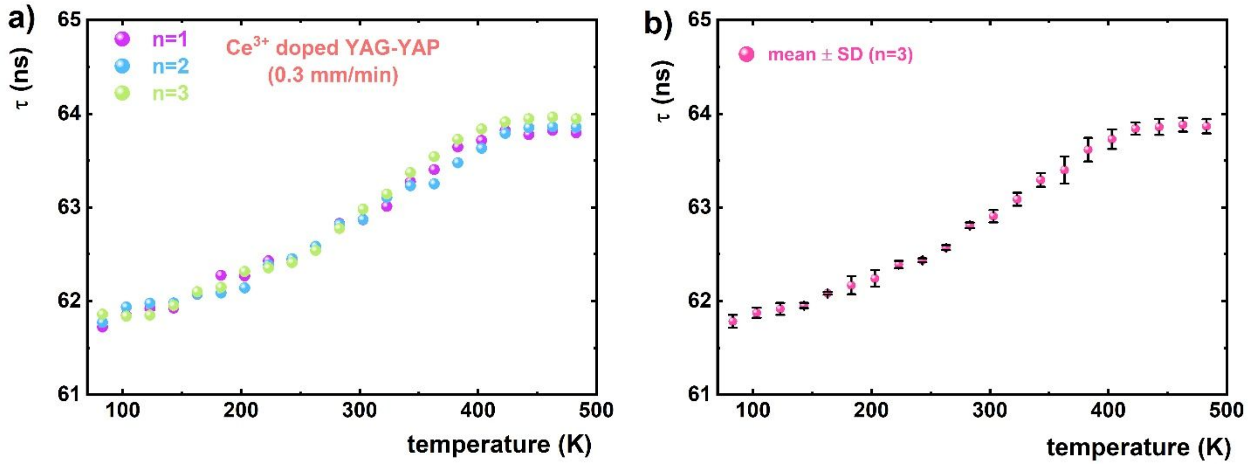

Figure S4. (a) Three independent, parallel temperature series (n = 1–3) of Ce^{3+} decay times (λ_{exc} = 460 nm, λ_{emi} = 570 nm) for Ce^{3+}-doped YAG-YAP eutetci crystal solidified at the rate of 0.3 mm/min measured under identical conditions. (b) Mean values at each temperature with error bars showing ±1 standard deviation (SD) from n = 3 replicates.

For each temperature T, three independent and identically performed experiments were conducted, and the results are presented as mean ± standard deviation (SD), see Figure S4a and S4b . The observed variability among replicates was consistently low across the entire temperature range, with a median SD of 0.069 ns. These values correspond to a median coefficient of variation (CV) of 0.11%, reflecting high precision of the experimental method. The largest replicate spread is found at T = 363 K, where the SD reached 0.146 ns (CV = 0.23%). Even at this maximum, the variation remains small relative to the signal magnitude, supporting the reliability of the measurement. The distinction between repeatability and reproducibility is important in interpreting these results. While reproducibility refers to the agreement of results obtained under varying conditions (such as different operators, instruments, or laboratories), the present study was designed to assess repeatability, that is, the agreement of results under identical conditions. The consistently narrow SD values observed confirm that the method yields highly repeatable results,

and suggest that it is also likely to perform well under broader reproducibility conditions, though this would require a separate, multi-variable study.

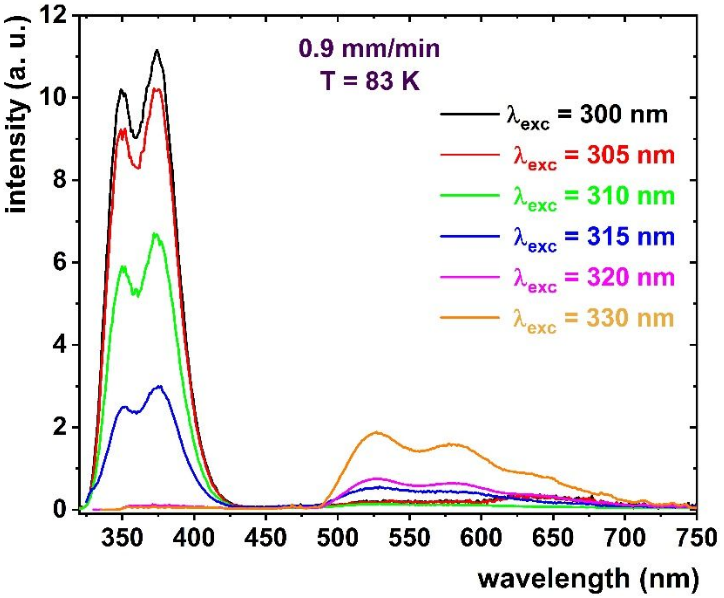

Figure S5. Photoluminescence emission spectra of the Ce^{3+}-doped eutectic crystal recorded at 83 K under various excitation wavelengths ranging from 300 to 330 nm. The PL emission centered at 375 nm is attributed to Ce^{3+} emission in the YAP phase, while the emission at 520 nm originates from Ce^{3+} in the YAG phase. The excitation wavelength of 315 nm yields the most balanced excitation of both luminescence centers.

Figure S5 presents the excitation-wavelength-dependent photoluminescence (PL) emission spectra of a eutectic crystal (0.9 mm/min) composed of Ce^{3+}-doped garnet (YAG) and perovskite (YAP) phases, recorded at a fixed temperature of 83 K. The excitation wavelength was varied over the range λ_{exc} = 300 – 330 nm to evaluate the selective and simultaneous excitation of Ce^{3+} ions in the YAP perovskite and YAG phases. At excitation wavelengths between 300 and 310 nm, the PL spectrum is dominated by the emission band centered at 375 nm, which is attributed to Ce^{3+} ions in the YAP phase. In this region, the emission from the garnet phase is negligible and insufficient to be considered as a second emissive component for ratiometric luminescence thermometry. This indicates that these higher-energy excitations do not efficiently excite the Ce^{3+} centers in the garnet phase. In contrast, excitation at 315 nm, which lies near the overlap of the excitation bands of Ce^{3+} in both garnet and perovskite phases, enables simultaneous excitation of both sites. Under this condition, both emission bands centered at 375 nm (YAP) and 520 nm (YAG), exhibit sufficient

intensity, making them suitable for reliable ratiometric analysis. Further increases in excitation wavelength (e.g., 320 and 325 nm) result in a marked decrease in the perovskite-related emission, indicating that these lower-energy photons are ineffective in exciting Ce^{3+} ions in the YAP lattice. Consequently, these wavelengths are not suitable for dual-site ratiometric thermometry. Based on this results, the 315 nm was selected as the optimal excitation wavelength for further studies, as it provides efficient and simultaneous excitation of Ce^{3+} ions in both the garnet and perovskite phases, thereby enabling accurate and sensitive ratiometric luminescent thermometry.

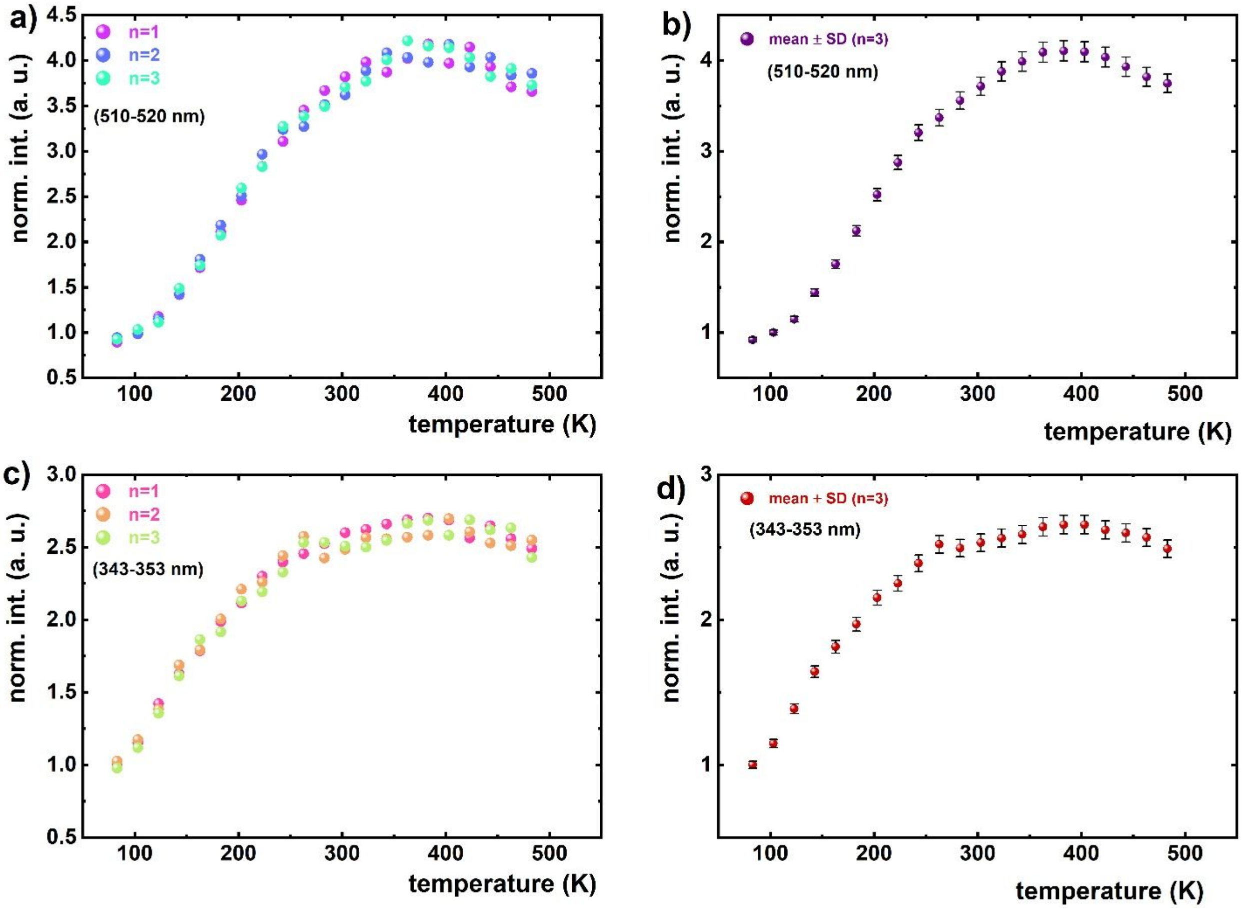

Figure S6. Evaluation of the repeatability of temperature-dependent luminescence intensity measurements for the emission bands centered at 510–520 nm (a, b) and 343–353 nm (c, d). Panels (a) and (c) show the raw normalized intensity data from three consecutive measurements (n = 1, 2, 3) performed on the same sample under identical experimental conditions. Panels (b) and (d) display the corresponding averaged curves with error bars representing standard deviation (SD) from the triplicate measurements at each temperature point. The consistently narrow SD across the entire temperature range confirms the high repeatability and precision of the measurement procedure.

To evaluate the repeatability and measurement precision of the luminescence response, temperature-dependent emission intensity measurements were performed on a Ce^{3+}-doped YAG–YAP eutectic crystal, solidified at a rate of 0.3 mm/min. At each temperature point, the eutectic crystal was measured three times consecutively under identical experimental conditions, without repositioning or modifying the setup. The results are presented in Figure S6. Panels (a) and (c) show the normalized intensity data from each of the three runs for the emission bands centered at 510–520 nm (YAG) and 343–353 nm (YAP), respectively. The good overlap of the data points across all three measurements confirms minimal run-to-run variability and excellent stability of the system. The corresponding averaged intensity curves are shown in panels (b) and (d), where error bars represent standard deviation (SD) from the triplicate measurements at each temperature. The SD values are consistently low throughout the entire temperature range (83 – 483 K), with a mean SD of 0.069 a.u. and a maximum of 0.146 a.u. at 363 K. These correspond to a coefficient of variation (CV) of 0.11%, and a maximum of 0.23%, both of which indicate high precision of the experimental procedure. This repeatability analysis demonstrates that the luminescence response of the Ce^{3+}-doped eutectic system is highly stable under fixed measurement conditions. The narrow SD values ensure that the observed temperature-dependent trends in emission intensity reflect intrinsic material behavior rather than instrumental fluctuations. This is important for applications in luminescence thermometry, where precise and reliable intensity readings are required to track temperature-induced variations in luminescence characteristics. Although the present measurements were designed to assess repeatability, defined as the agreement of results under identical conditions, the low observed dispersion suggests that the method is also inherently robust. While a full assessment of reproducibility (involving variations in sample batches, operators, instruments, and time) was not conducted here, the excellent agreement across repeated measurements suggest the reliability and stability of the optical setup and data acquisition protocol for Ce^{3+}-doped eutectic crystals prepared under controlled solidification conditions.

4. X-ray luminescence and thermally stimulated luminescence of eutectic crystals

Figure S7a compares the X-ray excited luminescence (XEL) spectra of Ce^{3+}-doped YAG-YAP eutectic crystals measured at 20 K. The spectra exhibit a dominant emission band in the range of 325-400 nm, attributed to the $5d_1 \rightarrow 4f$ radiative transition of Ce^{3+} ions in the YAP phase. At this

low temperature, the photoluminescence spectra display well-resolved characteristic doublet peaks, which correspond to the spin-orbit components of the $4f^1$ electronic configuration of Ce^{3+} ions: transitions from the $5d_1$ excited state to the ground-state doublet levels $^2F_{5/2}$ (shorter-wavelength band) and $^2F_{7/2}$ (longer-wavelength band) [3-6]. A low-intensity emission band between 480 and 650 nm corresponding to the $5d_1 \rightarrow 4f$ emission transition of Ce^{3+} ions within the YAG phase [2]. The emission lines observed at 386 nm and 416 nm are attributed to trace impurities of Tb^{3+} ions [7].

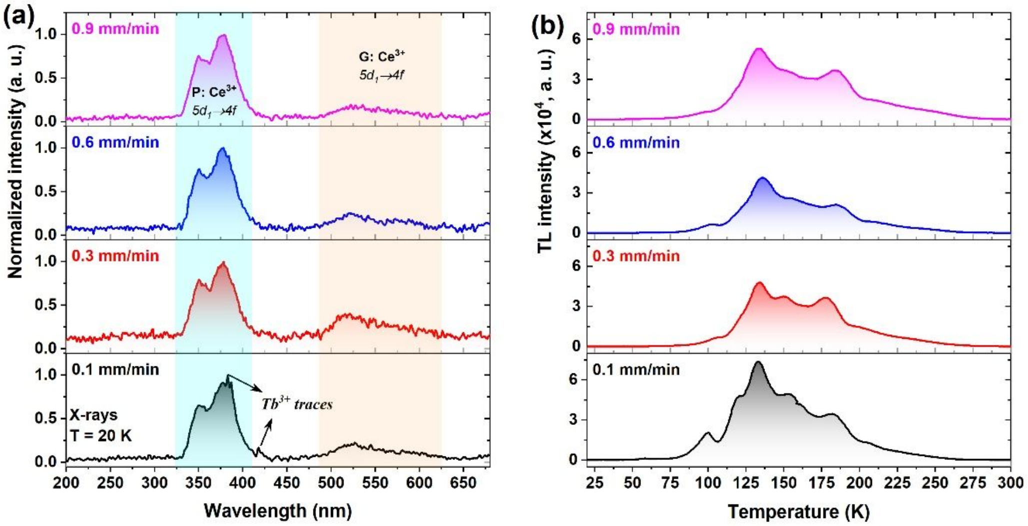

Figure S7. (a) X-ray excited luminescence spectra measured at 20 K and (b) thermoluminescence glow curves recorded between 20-300 K following X-ray irradiation at 20 K for Ce^{3+}-doped YAG-YAP eutectic crystals (0.1-0.9 mm/min).

The significantly lower intensity of this emission, relative to that of the perovskite phase, reflects the higher concentration of Ce^{3+} ions and volume fraction of the perovskite phase in the eutectic microstructure. This observation is consistent with the phase composition determined by powder X-ray diffraction patterns and EPMA analysis, which confirm the dominant presence of the YAP phase in these systems. Figure S7b compares the TL glow curve of all Ce^{3+}-doped YAG-YAP eutectic crystals. The TL glow curves exhibit a rich structure in the temperature range of 75-225 K, which is consistent across all samples. This feature is attributed to Y atoms occupying Al octahedral sites in both the garnet and perovskite phases, denoted as Y^x_{Al} dislocations [3, 8]. A notable decrease in TL intensity is observed for the samples solidified at higher rates. This suggests that

faster solidification limits the incorporation of Y atoms into Al octahedral sites, thereby reducing the concentration of Y^{x}_{Al} dislocations. The reduction in defect-related TL response implies improved structural order in rapidly solidified eutectic structures, likely due to restricted atomic mobility during solidification.

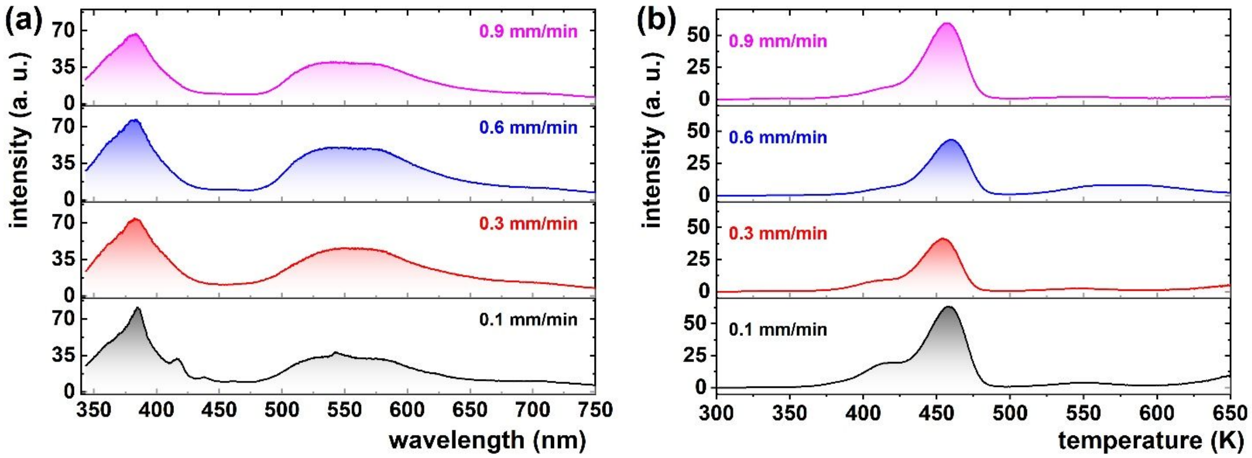

Figure S8. (a) X-ray excited luminescence spectra measured at 325 K and (b) thermoluminescence glow curves recorded between 300-714 K following X-ray irradiation at 300 K for Ce^{3+}-doped YAG-YAP eutectic crystals (0.1-0.9 mm/min).

Figure S8a presents the X-ray luminesce spectra measured at room temperature for Ce^{3+}-doped YAG-YAP eutectic crystals. The emission band centered at 375 nm is attributed to the Ce^{3+} ions emission in the YAP phase, while the broad emission centered around 550 nm is associated with the $5d \rightarrow 4f$ transitions of Ce^{3+} ions in the YAG phase. Additionally, narrow emission lines observed at approximately 386 nm, 417 nm, 438 nm and 545 nm are attributed to trace Tb^{3+} ion impurities. Figure S8b compares the TL glow curves of the same samples recorded over the 300-650 K temperature range. The TL profiles exhibit comparable peak shapes and intensities at approximately 410 K and 460 K across all eutectic crystals, indicating similar trap characteristics and recombination mechanisms. Notably, the sample crystallized at a rate of 0.6 mm/min displays a distinctly broadened and slightly more intense TL peak centered around 580 K. Those high-temperature features in all eutectics are attributed to deep trap levels, which are likely associated with oxygen vacancy-related defects and trace impurity centers [9, 10]. The comparable peak shapes and intensities of the peaks suggest that the crystallization rate has a negligible influence on the formation and stabilization of deep trapping states within the composite eutectic structures.

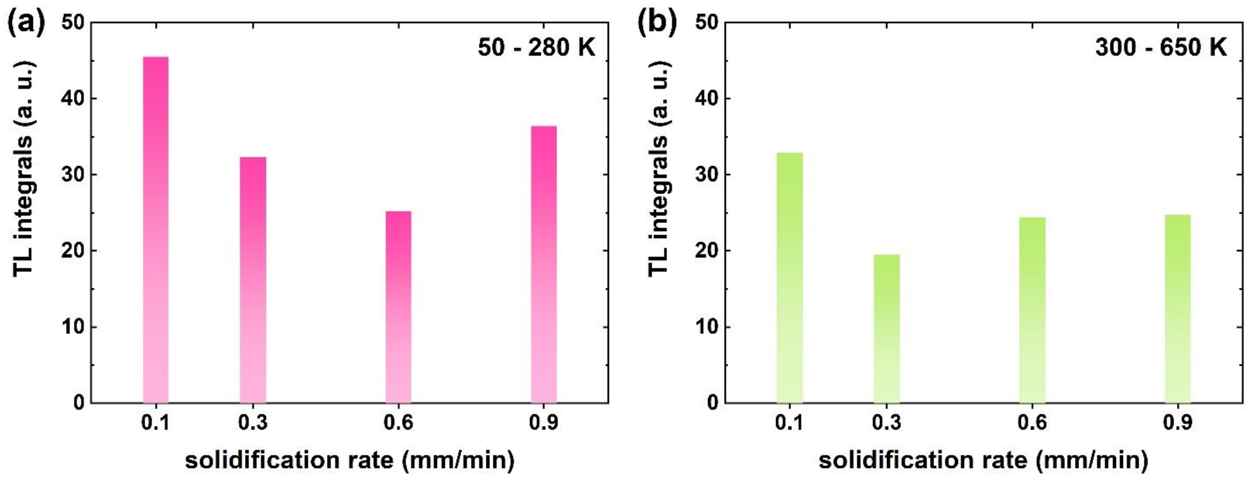

Figure S9. Integrated TL intensities between (a) 50 –280 K and (b) 300 - 650 K for Ce^{3+}-doped YAG-YAP eutectic crystals (0.1-0.9 mm/min).

The integrated TL intensities of Ce^{3+}-doped YAG-YAP eutectic crystals exhibit a clear dependence on the solidification rate, see Figure S9. In the low-temperature range (50–280 K, panel a), the TL integrals decrease from the highest value at 0.1 mm/min to a minimum at 0.6 mm/min, followed by a slight increase at 0.9 mm/min. A similar trend is observed in the high-temperature range (300–650 K, panel b), where TL intensity also decreases with increasing solidification rate from 0.1 mm/min to 0.3 mm/min, then stabilizes at 0.6 and 0.9 mm/min. In directionally solidified Ce^{3+}-doped YAG–YAP eutectic crystals, the lamellar phase distribution is highly sensitive to the solidification rate. The slowest solidification rate (e.g., 0.1 mm/min) results in coarse lamellae comprising YAG and YAP phase domains with dissimilar sizes on the order of sub-millimeters, whereas faster solidification rates (0.3–0.9 mm/min) produce a submicrometer-scale lamellar structure with similarly sized domains. This inverse relationship between pulling rate and interlamellar spacing is consistent with eutectic solidification theory (Jackson–Hunt model) and has been observed in analogous systems [11, 12]. The present results show that morphology of the eutectic crystals, especially the size of the YAG and YAP phases and the lamellar spacing (i.e. density of YAG-YAP phase boundaries) have a marked effect on point defect formation. The TL intensity associated with Y^{x}_{Al} displacement (antisite defects) and oxygen

vacancy ($V^{\bullet\bullet}_O$) traps decreases markedly in the fast-grown eutectics with sub-micrometer interlamellar spacing, indicating a lower concentration of these defects compared to the slowly grown (coarse-lamellar) eutectics. This correlation suggests that the phase distribution (lamellar spacing and interface area) directly influences the formation and retention of intrinsic defects in the composite eutectics. Notably, this is the first study to directly examine the microstructure–defect relationship in the Ce^{3+}-doped YAG–YAP eutectic system, therefore, the understanding relies on established defect physics in garnet and perovskite phases.

The reduction in Y^x_{Al} displacement and oxygen-vacancy traps at higher solidification rates arises from the interplay between defect-formation energetics and crystallization kinetics. In YAG and YAP, the Y^x_{Al} displacement is the lowest-energy intrinsic point defect [3, 13-15]. Such Y^x_{Al} displacement are well-known to act as charge carrier traps and luminescence quenchers in aluminium garnets and perovskites [6, 16-18]. The Y^x_{Al} displacements, in a relatively high concentration, are formed in the aluminum garnet compounds due to high temperatures during the crystal growth from the melt [9, 19-22]. The oxygen vacancies are prevalent intrinsic defects in YAG and YAP under oxygen-deficient conditions, becoming energetically favorable when the chemical potential of oxygen is low (e.g. in the inert environment) [3, 9, 18, 23]. . Both types of point defects (Y^x_{Al} and $V^{\bullet\bullet}_O$) create shallow and deep levels in the bandgap and are responsible for characteristic TL glove curve between 50 -200 K (Y^x_A) and over 300 K ($V^{\bullet\bullet}_O$) [3, 9, 18]. The actual density of these defects in a crystal is determined not only by their formation energies (thermodynamics) but also by the kinetics of the solidification and cooling process. Slower solidification (formation of sub-millimeter lamellae morphology with dissimilar sizes) effectively means the material spends a longer time at high temperature in near-equilibrium conditions, which allows cation sublattices to equilibrate and defects to form or migrate toward their equilibrium positions. In a slowly grown eutectic, Y and Al atoms have more time to swap sites or deviate locally to relieve any slight compositional imbalances between the coexisting YAG and YAP phases. As a result, a significant equilibrium concentration of Y^x_A displacement can be established [13]. Similarly, a slower solidification rate can facilitate the formation and aggregation of oxygen vacancies, particularly under inert atmospheric conditions during crystal growth. At elevated temperatures, the stabilization of these vacancies is further enhanced by the partial oxidation of Ce^{3+} to Ce^{4+}, which induces local charge imbalance and promotes vacancy retention within the crystal lattice [4]. Oxygen, being relatively mobile at the melting/solidification temperature, may diffuse out of the

lattice (or into sinks such as interfaces) during slow cooling, leaving behind vacancies. In contrast, faster solidification, characterized by the formation of sub-micrometer-scale lamellar morphologies with similar sizes of YAG and YAP phases significantly reduces the time available for defect formation and atomic rearrangement. Under these conditions, the rapidly advancing solid–liquid interface can result in solute trapping, whereby constituent elements are incorporated into the solid phase in (near)stoichiometric proportions, rather than undergoing complete compositional partitioning and site ordering as would occur under slow solidification conditions [24, 25]. This means Y^{3+} and Al^{3+} ions are more likely to occupy their sublattice sites as there is insufficient time for the long-range diffusion or site-exchange events. This mechanism is supported by the improved segregation behavior of Ce^{3+} ions. As the solidification rate increases, the distribution of Ce^{3+} ions become more homogeneous, approaching nearly uniformity in eutectic solidified at a rate of 0.9 mm/min. This suggests that at higher solidification rates, Ce^{3+} ions have insufficient time to segregate toward the crystal rim, a phenomenon observed in eutectic crystals solidified at the slower rate of 0.1 mm/min [23, 26]. Furthermore, reduction of lamellar spacing to the sub-micrometer scale markedly increases the density of YAG–YAP phase boundaries, which serve as effective sinks for point defects during the cooling process. In such submicron periodic morphology, Y^{x}_{Al} displacement and oxygen vacancies remain in close proximity to heterophase interfaces, whose characteristic strain fields and dangling bonds are capable of attracting and trapping these defects, thereby facilitating their removal or deactivation within the bulk lattice. In perovskite-related oxides, lattice strain has been demonstrated to modulate oxygen vacancy formation energies [27], thus, the considerable strain present at YAG–YAP interfaces may further elevate the energetic barrier for vacancy formation in the contiguous bulk, indirectly suppressing the creation of oxygen vacancies. Moreover, rapid cooling (i.e. quick solidification rate) substantially limits the diffusion of oxygen from the crystal lattice, as the quenching process precedes significant oxygen loss. Consequently, the resultant phase exhibits a more completely oxidized structure with a diminished concentration of oxygen vacancies.

5. *Characteristics of undoped YAG-YAP eutectic crystal (0.7 mm/min)*

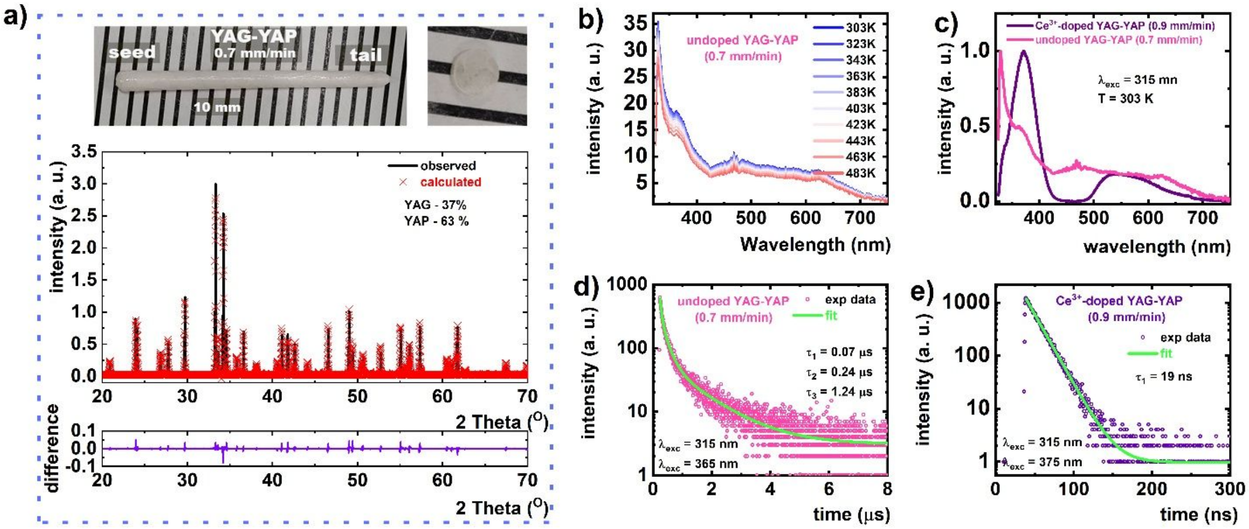

Figure S10. (a) Photograph of the undoped YAG–YAP eutectic crystal grown by the micro-pulling-down method at a solidification rate of 0.7 mm/min (upper panel); X-ray diffraction (XRD) pattern of the eutectic sample with Rietveld refinement. The measured (black line) and calculated (red crosses) profiles exhibit agreement, with the difference plot shown below. Phase composition determined from the refinement indicates 37% YAG and 63% YAP by volume (lower panle). (b) Photoluminescence (PL) emission spectra of undoped YAG-YAP (grown at 0.7 mm/min) recorded under UV excitation (λ_{exc} = 315 nm) across a temperature range of 303-483 K. (c) Comparison of RT PL spectra between undoped and Ce^{3+}-doped YAG-YAP crystals excited at 315 nm. (c) PL decay curve of undoped YAG-YAP (0.7 mm/min) for defect related emission at 365 nm under excitation at 315 nm. (d) PL decay curve of Ce^{3+}-doped YAG-YAP for Ce^{3+} emission at 375 nm under excitation at 315 nm.

Figure S10a displays a photograph of the as-grown, undoped YAG-YAP eutectic crystal, solidified at a rate of 0.7 mm/min, alongside the corresponding polished cylindrical sample. The eutectic crystal exhibits white coloration attributable to light scattering at the phase boundaries between YAG and YAP, thereby confirming the successful formation of a homogeneous eutectic rod with well-defined geometry. X-ray diffraction (XRD) analysis, supported by Rietveld refinement, reveals that the sample contains two crystalline phases YAG (37%) and YAP (63%). Figure S10b presents the temperature-dependent PL spectra of the undoped YAG-YAP eutectic crystal in the 303–463 K range under 315 mn excitation. A broad, structureless emission band centered around 350–420 nm is observed at room temperature, with its intensity gradually decreasing with increasing temperature. This thermal quenching behavior is characteristic of defect-related luminescence, where non-radiative recombination becomes more prominent at elevated temperatures [28]. The lack of fine structure further supports the attribution of this emission to

intrinsic lattice defects rather than to transitions of optically active dopant ions. To differentiate this defect-related emission from Ce^{3+} dopants, Figure S10c compares the PL spectra of undoped and Ce^{3+}-doped YAG-YAP crystals excited at 315 nm and measured at 303 K. The Ce^{3+}-doped sample displays two intense emission bands in the 370–500 nm range, corresponding to the allowed 5d → 4f transitions of Ce^{3+} ions in the YAP and YAG phases. In contrast, the undoped sample exhibits a broader emission profile without distinct features, supporting the conclusion that its luminescence originates from defect-related centers and not from Ce^{3+} ions. The photoluminescence decay time measurements further support these findings. As shown in Figure S10d, the PL decay time for emission at 365 nm of the undoped YAG-YAP crystal under 315 nm excitation follows a tri-exponential decay behavior with lifetimes of τ_1 = 0.07 μs, τ_2 = 0.24 μs, and τ_3 = 1.24 μs. These relatively long and distributed decay components are consistent with carrier recombination via multiple defect states with varying trap depths and activation energies [22, 29]. Such decay dynamics are typical of disordered systems where charge carriers are temporarily localized at defects before recombination. In contrast, the Ce^{3+}-doped YAG-YAP crystal exhibits a markedly different PL decay time profile (Figure S10e). Under excitation at 315 and monitoring emission at 375 nm, the decay is shows a single-exponential function with a decay time of τ = 19 ns, consistent with the decay of Ce^{3+} ions in the YAP phase.

Undoped YAG–YAP eutectic crystal exhibits a broad ultraviolet emission band in the 320–420 nm range under 315 nm excitation. This emission is attributed to intrinsic point defects in the host lattice, particularly oxygen vacancies (F-centers and F^+ centers) and antisite defects, where Y^{3+} ions occupy Al^{3+} sites (Y^x_{Al})[30, 31]. These defects introduce localized electronic states within the bandgap, which can trap charge carriers and excitons act as radiative recombination centers. Specifically, oxygen-vacancy-related centers and excitons bound to antisite sites give UV-blue luminescence in both YAG and YAP systems, with emissions in the ~2.75–4.1 eV range (~300–450 nm) being commonly associated with such defect-related processes [21, 22, 32]. As temperature increases, the intensity of this defect-related luminescence diminishes significantly, a phenomenon typically attributed to thermal quenching. With increasing thermal energy, charge carriers trapped at shallow defect sites gain sufficient energy to escape into the conduction band or migrate to non-radiative centers, where they undergo phonon-assisted relaxation [31, 33]. This de-trapping process reduces the probability of radiative recombination at the defect site, leading to a decrease in UV emission. The suppression of luminescence at temperatures approaching ~483 K is therefore

consistent with shallow trap-related recombination mechanisms, where radiative efficiency decreases due to enhanced non-radiative pathways. In contrast, Ce^{3+}-doped YAG–YAP eutectic crystals exhibit a markedly different temperature dependence. Under the same excitation wavelength (315 nm), the Ce^{3+} emission intensity increases with rising temperature. This emission, centered in the UV (YAP) and visible range (YAG), originates from the allowed $5d_1 \rightarrow 4f$ transitions of Ce^{3+} ions [31, 32]. The thermally enhanced Ce^{3+} luminescence can be explained by trap-mediated energy transfer mechanisms. At low temperatures, the excitation energy is lost through non-radiative recombination at intrinsic defect centers or becomes trapped in long-lived, non-emission states. As temperature increases, carriers trapped at shallow defects are thermally released and can be captured by nearby Ce^{3+} ions, thereby populating their excited states and enhancing visible emission [9]. Additionally, Ce^{3+} doping itself can influence the defect structure of the host lattice, potentially introducing charge-compensating vacancies or modifying the density of shallow traps [31]. This can further promote thermal charge carrier mobility and enhance the likelihood of energy transfer from defect states to Ce^{3+} activators. The inverse thermal behavior, i.e., decreasing defect-related emission and increasing Ce^{3+} luminescence reveals a dynamic shift in recombination pathways as temperature rises. At lower temperatures, defect centers dominate the recombination process, but with increasing thermal activation, the recombination channel is progressively redirected toward Ce^{3+} luminescent centers. This results in the increase in Ce^{3+} emission and a reduction in UV defect-related emission.

Funding Sources

This project has received funding from the European Union's Horizon Europe research and innovation programme under the Marie Skłodowska-Curie Actions COFUND, Physics for Future, grant agreement no. 101081515 and by the GIMRT Program of the Institute for Materials Research, Tohoku University, proposal No. 202412-RDKYA-0503. M. S. gratefully acknowledges the support of the Foundation for Polish Science through the START program. The work is supported by OP JAC financed by ESIF and the MEYS SENDISO - CZ.02.01.01/00/22_008/0004596. Part of the thermoluminescence and radioluminescence results was supported by funding from the National Science Centre, Poland, under project number 2023/49/B/ST5/04265 JZ and AO gratefully acknowledge this support. Financial support for the Postdoctoral fellowship project of the Research Council of Lithuania (No. S-PD-24-63).

References

(1) Zhou, W.; Lou, C.; Zhou, W.; Lou, C. Fabrication of YAG:Ce^{3+} and YAG:Ce^{3+}, Sc^{3+} Phosphors by Spark Plasma Sintering Technique. *Journal of Wuhan University of Technology* **2024**, *39* (2). DOI: 10.1007/s11595-024-2878-6.

(2) Ikesue, A.; Aung, Y. L.; Honda, S.; Iwamoto, Y. Performance of Ce:YAG-MgO composite ceramics as high-power LED phosphor densified by non-reactive sintering. *Journal of the European Ceramic Society* **2024**, *44* (8), 5235-5240. DOI: 10.1016/j.jeurceramsoc.2024.02.056.

(3) Bartosiewicz, K.; Pejchal, J.; Kucerkova, R.; Beitlerova, A.; Babin, V.; Vanecek, V.; Kurosawa, S.; Kamada, K.; Yoshikawa, A. Advances in Ce^{3+} doped $Y_{1\pm x}AlO_3$ ($x\neq0$) single crystal perovskite scintillators through nonstoichiometric engineering. *Optical Materials: X* **2024**, 100295-100295. DOI: 10.1016/j.omx.2024.100295.

(4) Bartosiewicz, K.; Szysiak, A.; Tomala, R.; Gołębiewski, P.; Węglarz, H.; Nagirnyi, V.; Kirm, M.; Romet, I.; Buryi, M.; Jary, V.; et al. Energy-Transfer Processes in Nonstoichiometric and Stoichiometric Er^{3+}, Ho^{3+}, Nd^{3+}, Pr^{3+}, and Cr^{3+}-Codoped Ce:YAG Transparent Ceramics: Toward High-Power and Warm-White Laser Diodes and LEDs. *Physical Review Applied* **2023**, *20* (1). DOI: 10.1103/PhysRevApplied.20.014047.

(5) Bartosiewicz, K.; Fritz, V.; Van, D.; Szymański, D.; Justyna, Z.; Akihiro, Y.; Pejchal, J.; Kučerková, R.; Beitlerová, A.; Kurosawa, S.; et al. Towards deliberate design of persistent phosphors: a study of La–Ga admixing in LuAG:Ce crystals to engineer elemental homogeneity and carrier trap depths. *Journal of Materials Chemistry C* **2023**, *11* (26), 8850-8865. DOI: 10.1039/d3tc01304a (acccessed 2024).

(6) Baccaro, S.; Blazek, K.; Denotaristefani, F.; Maly, P.; Mares, J. A.; Pani, R.; Pellegrini, R.; Soluri, A. Scintillation Properties of YAP:Ce. *Nucl Instrum Meth A* **1995**, *361* (1-2), 209-215. DOI: 10.1016/0168-9002(95)00016-X.

(7) Bartosiewicz, K.; Babin, V.; Beitlerova, A.; Bohacek, P.; Jurek, K.; Nikl, M. The temperature dependence studies of rare-earth (Dy^{3+}, Sm^{3+}, Eu^{3+} and Tb^{3+}) activated $Gd_3Ga_3Al_2O_{12}$ garnet single crystals. *J Lumin* **2017**, *189*, 126-139. DOI: 10.1016/j.jlumin.2016.09.053.

(8) Omuro, K.; Yoshino, M.; Bartosiewicz, K.; Horiai, T.; Murakami, R.; Kim, K. J.; Kamada, K.; Kucerkova, R.; Babin, V.; Nikl, M.; et al. Insights into luminescence and energy transfer processes in Ce^{3+}- and Tb^{3+} co-doped $(Gd,Y)_3Al_2Ga_3O_{12}$ garnet single crystals. *J Lumin* **2024**, *273*, 120663. DOI: 10.1016/j.jlumin.2024.120663.

(9) Bartosiewicz, K.; Smortsova, Y.; Radmoski, P.; Witkowski, M. E.; Drozdowski, K. J.; Yoshino, M.; Horiai, T.; Szymański, D.; Dewo, W.; Zeler, J.; et al. Shaping scintillation and UV-VIS-NIR luminescence properties through synergistic lattice disordered engineering and exciton-mediated energy transfer in Pr^{3+}-doped $Lu_{1.5}Y_{1.5}Al_{5-x}Sc_xO_{12}$ ($x = 0.0$–2.0) garnets. *Journal of Materials Chemistry C* **2025**, *13* (27), 13691-13712. DOI: 10.1039/d5tc01411e.

(10) Khanin, V. M.; Venevtsev, I.; Chernenko, K.; Tukhvatulina, T.; Rodnyi, P. A.; Spoor, S.; Boerekamp, J.; van Dongen, A.-M.; Buettner, D.; Wieczorek, H.; et al. Influence of 3d Transition Metal Impurities on Garnet Scintillator Afterglow. *Crystal Growth & Design* **2020**, *20* (5), 3007-3017. DOI: 10.1021/acs.cgd.9b01660.

(11) Ludwig, A.; Leibbrandt, S. Generalised 'Jackson-Hunt' model for eutectic solidification at low and large Peclet numbers and any binary eutectic phase diagram. *Mat Sci Eng a-Struct* **2004**, *375*, 540-546. DOI: 10.1016/j.msea.2003.10.108.

(12) Yasuda, H.; Ohnaka, I.; Mizutani, Y.; Sugiyama, A.; Morikawa, T.; Takeshima, S.; Sakimura, T.; Waku, Y. Solidification and shape casting of Al_2O_3–YAG eutectic ceramics from the undercooled melt produced by melting Al_2O_3–YAP eutectics. *Science and Technology of Advanced Materials* **2004**, *5* (1-2), 207-217. DOI: 10.1016/j.stam.2003.10.022.

(13) Liu, B.; Gu, M.; Liu, X. L.; Huang, S. M.; Ni, C. Formation energies of antisite defects in $Y_3Al_5O_{12}$: A first-principles study. *Applied Physics Letters* **2009**, *94* (12), 121910. DOI: 10.1063/1.3109799.

(14) Vedda, A.; Martini, M.; Meinardi, F.; Chval, J.; Dusek, M.; Mares, J. A.; Mihokova, E.; Nikl, M. Tunneling process in thermally stimulated luminescence of mixed $Lu_xY_{1-x}AlO_3$:Ce crystals. *Physical Review B* **2000**, *61* (12). DOI: 10.1103/PhysRevB.61.8081.
(15) Kuklja, M. M. Defects in yttrium aluminium perovskite and garnet crystals: atomistic study. *Journal of Physics: Condensed Matter* **200**, *12* (13), 2953-2967. DOI: 10.1088/0953-8984/12/13/307.
(16) Krasnikov, A.; Savikhina, T.; Zazubovich, S.; Nikl, M.; Mares, J. A.; Blazek, K.; Nejezchleb, K. Luminescence and defects creation in Ce^{3+}-doped aluminium and lutetium perovskites and garnets. *Nuclear Instruments and Methods in Physics Research Section A: Accelerators, Spectrometers, Detectors and Associated Equipment* **2005/01/21**, *537* (1-2), 130-133. DOI: 10.1016/j.nima.2004.07.251.
(17) Korzhik, M.; Gola, A.; Houzvicka, J.; Mazzi, A.; Nargelas, S.; Skorová, S.; Tamulaitis, G.; Vaitkevicius, A. Timing properties of Ce-doped YAP and LuYAP scintillation crystals. *Nucl Instrum Meth A* **2019**, *927*, 169-173. DOI: 10.1016/j.nima.2019.02.036.
(18) Nikl, M.; Yoshikawa, A.; Kamada, K.; Nejezchleb, K.; Stanek, C. R.; Mares, J. A.; Blazek, K. Development of LuAG-based scintillator crystals – A review. *Progress in Crystal Growth and Characterization of Materials* **2013**, *59* (2), 47-72. DOI: 10.1016/j.pcrysgrow.2013.02.001.
(19) Blazek, K.; Krasnikov, A.; Nejezchleb, K.; Nikl, M.; Savikhina, T.; Zazubovich, S. Luminescence and defects creation in Ce^{3+}-doped $Lu_3Al_5O_{12}$ crystals. *Phys Status Solidi B* **2004**, *241* (5), 1134-1140. DOI: 10.1002/pssb.200301986.
(20) Nikl, M.; Vedda, A.; Fasoli, M.; Fontana, I.; Laguta, V. V.; Mihokova, E.; Pejchal, J.; Rosa, J.; Nejezchleb, K. Shallow traps and radiative recombination processes in $Lu_3Al_5O_{12}$:Ce single crystal scintillator. *Physical Review B* **2007**, *76* (19). DOI: 10.1103/PhysRevB.76.195121.
(21) Bartosiewicz, K.; Horiai, T.; Yamaji, A.; Yoshikawa, A.; Kurosawa, S.; Kim, K. J.; Vistovskyy, V.; Voloshinovskii, A.; Zorenko, Y. Bright exciton luminescence from La doped $Lu_3Al_5O_{12}$ single crystals. *J Lumin* **2021**, *235*. DOI: 10.1016/j.jlumin.2021.118013.
(22) Bartosiewicz, K.; Horiai, T.; Yamaji, A.; Yoshikawa, A.; Kurosawa, S.; Yoshino, M.; Zorenko, Y. Effects of La doping on the crystal growth, phase stability and scintillation properties of $Lu_3Al_5O_{12}$ single crystals. *Mater Sci Eng B-Adv* **2020**, *261*. DOI: 10.1016/j.mseb.2020.114677.
(23) Bartosiewicz, K.; Dewo, W.; Nagirnyi, V.; Runka, T.; Kirm, M.; Horiai, T.; Szymanski, D.; Yamaji, A.; Kurosawa, S.; Socha, P.; et al. Correlating Structural Disorder and Pr^{3+} Emission Dynamics in $Lu_3Al_{2.5-x}Sc_xGa_{2.5}O_{12}$ Crystals: A Comprehensive Structure–Property Investigation. *ACS Omega* **2025**, *10* (19), 19817-19831. DOI: 10.1021/acsomega.5c01062.
(24) Orera, V. M.; Merino, R. I.; Pardo, J. A.; Larrea, A.; Peña, J. I.; González, C.; Poza, P.; Pastor, J. Y.; Llorca, J. Microstructure and physical properties of some oxide eutectic composites processed by directional solidification. *Acta Materialia* **2000**, *48* (18-19), 4683-4689. DOI: 10.1016/S1359-6454(00)00258-5.
(25) Liu, H. F.; Su, H. J.; Shen, Z. L.; Jiang, H.; Zhao, D.; Liu, Y.; Guo, Y. N.; Li, X.; Guo, M.; Zhang, J.; et al. Formation mechanism and roles of oxygen vacancies in melt-grown $Al_2O_3/GdAlO_3/ZrO_2$ eutectic ceramic by laser 3D printing. *Journal of Advanced Ceramics* **2022**, *11* (11), 1751-1763. DOI: 10.1007/s40145-022-0645-8.
(26) Bartosiewicz, K.; Albini, B.; Szymański, D.; Socha, P.; Horiai, T.; Yoshino, M.; Yamaji, A.; Kurosawa, S.; Kucerkova, R.; Galinetto, P.; et al. Engineering atomic size mismatch in Pr^{3+}, La^{3+} codoped $Lu_3Al_5O_{12}$ garnet single crystals for tailored structure and functional properties. *J Alloy Compd* **2024**, *985*. DOI: 10.1016/j.jallcom.2024.174078.
(27) Li, M.; Li, J.; Chen, L.-Q.; Gu, B.-L.; Duan, W. Effects of strain and oxygen vacancies on the ferroelectric and antiferrodistortive distortions in $PbTiO_3/SrTiO_3$ superlattice. *Physical Review B* **2015**, *92* (11), 115435. DOI: 10.1103/PhysRevB.92.115435.
(28) Laguta, V.; Buryi, M.; Arhipov, P.; Sidletskiy, O.; Laguta, O.; Brik, M. G.; Nikl, M. Oxygen-vacancy centers in $Y_3Al_5O_{12}$ garnet crystals: electron paramagnetic resonance and dielectric spectroscopy study. *Physical Review B* **2020**, *101* (2), 024106. DOI: 10.1103/PhysRevB.101.024106.

(29) Bartosiewicz, K.; Babin, V.; Kamada, K.; Yoshikawa, A.; Beitlerova, A.; Nikl, M. Effects of Gd/Lu ratio on the luminescence properties and garnet phase stability of Ce^{3+} activated $Gd_xLu_{3-x}Al_5O_{12}$ single crystals. *Opt Mater* **2018**, *80*, 98-105. DOI: 10.1016/j.optmat.2018.04.023.
(30) Zych, E.; Brecher, C.; Wojtowicz, A. J.; Lingertat, H. Luminescence properties of Ce-activated YAG optical ceramic scintillator materials. *J Lumin* **1997**, *75* (3), 193-203. DOI: 10.1016/S0022-2313(97)00103-8.
(31) Laguta, V.; Buryi, M.; Babin; Machek, P.; Zazubovich, S.; Bartosiewicz, K.; Kurosawa, S.; Yamaji, A.; Yoshikawa, A.; Ulicná, K.; et al. Li^+ incorporation and defect-creation processes imposed by X-ray and UV irradiation in Li-codoped $Y_3Al_5O_{12}$:Ce scintillation crystals. *Journal of Materials Chemistry C* **2023**, *11* (4), 1346-1359. DOI: 10.1039/d2tc04520f.
(32) Nikl, M.; Yoshikawa, A. Recent R&D Trends in Inorganic Single-Crystal Scintillator Materials for Radiation Detection. *Adv Opt Mater* **2015**, *3* (4), 463-481. DOI: 10.1002/adom.201400571.
(33) Laguta, V.; Buryi, M.; Arhipov, P.; Sidletskiy, O.; Laguta, O.; Brik, M. G.; Nikl, M. Oxygen-vacancy donor-electron center in $Y_3Al_5O_{12}$ garnet crystals: Electron paramagnetic resonance and dielectric spectroscopy study. *Physical Review B* **2020**, *101* (2). DOI: 10.1103/PhysRevB.101.024106.
